# Breed differences in social cognition, inhibitory control, and spatial problem-solving ability in the domestic dog (*Canis familiaris*)

**DOI:** 10.1038/s41598-022-26991-5

**Published:** 2022-12-29

**Authors:** Saara Junttila, Anna Valros, Katariina Mäki, Heli Väätäjä, Elisa Reunanen, Katriina Tiira

**Affiliations:** 1 Department of Production Animal Medicine, University of Helsinki, 00014 Helsinki, Finland; 2 International Partnership for Dogs, Helsinki, Finland; 3 Digital Solutions, Lapland University of Applied Sciences, Jokiväylä 11C, 96300 Rovaniemi, Finland; 4 Department of Finnish and Finno-Ugric Languages, University of Turku, 20014 Turku, Finland; 5 smartDOG Ltd, 05800 Hyvinkää, Finland; 6 Department of Equine and Small Animal Medicine, University of Helsinki, 00014 Helsinki, Finland

**Keywords:** Learning and memory, Social behaviour, Animal behaviour

## Abstract

The extraordinary genetic and behavioural diversity of dog breeds provides a unique opportunity for investigating the heritability of cognitive traits, such as problem-solving ability, social cognition, inhibitory control, and memory. Previous studies have mainly investigated cognitive differences between breed groups, and information on individual dog breeds is scarce. As a result, findings are often contradictory and inconsistent. The aim of this study was to provide more clarity on between-breed differences of cognitive traits in dogs. We examined the performance of 13 dog breeds (N = 1002 dogs) in a standardized test battery. Significant breed differences were found for understanding of human communicative gestures, following a human’s misleading gesture, spatial problem-solving ability in a V-detour task, inhibitory control in a cylinder test, and persistence and human-directed behaviour during an unsolvable task. Breeds also differed significantly in their behaviour towards an unfamiliar person, activity level, and exploration of a novel environment. No significant differences were identified in tasks measuring memory or logical reasoning. Breed differences thus emerged mainly in tasks measuring social cognition, problem-solving, and inhibitory control. Our results suggest that these traits may have come under diversifying artificial selection in different breeds. These results provide a deeper understanding on breed-specific traits in dogs.

## Introduction

Cognitive abilities (such as learning, memory, inhibitory control, problem-solving, and social cognition) are important traits in almost all aspects of an animal’s life, from finding food to cooperating with conspecifics. Despite this, the heritability of cognitive traits in non-human animals is still a largely unknown topic^1^. The domestic dog (*Canis familiaris*), with its extraordinary genetic and phenotypic diversity, provides a unique opportunity for advancing our understanding of this subject. Behavioural variation between dog breeds is substantial^2^, and there is evidence that many of these breed differences are attributable to genetic factors^3^ (but see Morrill et al.^4^).

Cognitive differences between breeds have been investigated to some extent, but the results seem to be largely contradictory and inconsistent. For example, studies have demonstrated differences in breeds’ understanding of human referential cues, such as pointing or gazing^5–8^, and genetic relatedness between breeds seems to account for a substantial portion of variation in this trait^9^. Several other studies, however, have not been able to replicate these results^10–13^. Similarly, studies have failed to find any significant differences between breeds for logical reasoning ability^13^, inhibitory control^13^, or memory^12,13^, although Gnanadesikan et al.^9^ demonstrated that genetic relatedness among breeds accounted for a significant proportion of variation in these traits.

The difficulty in interpreting previous results lies partly in the fact that most studies have grouped breeds together, either based on original function or genetic relatedness. What further complicates interpretation is that across studies, groups are often composed of different breeds, and the criteria for categorizing breeds differ greatly. Some authors have questioned the use of breed group classifications based on the breeds’ original purpose^14–17^, mainly because selection pressures may have changed drastically. For instance, Svartberg^17^ found no associations between behavioural profiles of breeds and their original purpose; instead, breed-typical behaviour was correlated with the current use of the breed. Even grouping breeds based on their genetic relatedness is not without its faults. For example, both Svartberg^17^ and Turcsán et al.^16^ used cluster analysis to group breeds based on behavioural similarity, and found that these clusters corresponded poorly with the genetic categorization of breeds. These results suggest that when breeds are divided into groups, important differences between breeds may be missed.

Only a handful of studies have compared cognitive differences between individual breeds instead of breed groups^7,8,12,14,18–20^. Unfortunately, most of these have involved restricted sample sizes and a limited number of breeds. Moreover, very little empirical research has targeted non-social cognitive traits such as memory, inhibitory control, spatial problem-solving, and logical reasoning. The aim of our study was to provide a more complete picture of cognitive differences between dog breeds. We explored breed differences not only in dogs’ socio-cognitive abilities, but also in several cognitive traits not involving a social aspect. In addition, we compared breeds regarding their exploration in a novel space, greeting an unfamiliar person, and activity level, since these may also be linked to cognitive performance^21–24^. Our large sample size allowed us to look at differences between individual breeds rather than having to resort to breed group classifications. Our aim was to provide a more comprehensive understanding of how breeds vary cognitively and behaviourally, which improves our ability to predict how individual dogs are likely to behave. Our results may also further our understanding of the heritability and genetics of cognition, both in humans and in other animals.

## Methods

### Subjects

A total of 2,352 adult dogs participated in a smartDOG™ test battery^25^ between March 2016 and February 2022. Participating dogs were required to be interested in working for food, and to not be overly aggressive to people. We limited the analysis to dogs between the ages of 1 and 8 years, since cognitive traits may not have fully developed in younger dogs^26,27^, while older dogs may experience cognitive decline^28^. We included only dog breeds with a minimum of 40 individuals tested per breed. This resulted in a final sample size of 1,002 dogs representing 13 breeds, including one category consisting of mixed breeds. Any individual which had parents belonging to different breeds was classified as mixed breed, except for the Labradoodle. Details of participant dogs’ ages, sexes, and breeds are presented in Table 1.

**Table 1:** Number of dogs within each breed and their sexes and median ages.

Breed	n	Males (%)	Females (%)	Median age in years ± IQR
Australian Kelpie	41	20 (48.8)	21 (51.2)	3.0 ± 2.8
Australian Shepherd	49	23 (46.9)	26 (53.1)	2.9 ± 2.6
Belgian Shepherd Malinois	49	34 (69.4)	15 (30.6)	3.6 ± 2.6
Border Collie	106	48 (45.3)	58 (54.7)	2.6 ± 2.3
English Cocker Spaniel	60	29 (48.3)	31 (51.7)	2.7 ± 2.7
Finnish Lapphund	59	28 (47.5)	31 (52.5)	2.8 ± 3.4
German Shepherd	82	34 (41.5)	48 (58.5)	2.6 ± 2.9
Golden Retriever	74	35 (47.3)	39 (52.7)	2.8 ± 2.5
Hovawart	50	23 (46.0)	27 (54.0)	2.7 ± 2.9
Labrador Retriever	163	69 (42.3)	94 (57.7)	2.6 ± 2.3
Mixed Breed	149	54 (36.2)	95 (63.8)	3.3 ± 2.8
Shetland Sheepdog	48	19 (40.4)	28 (59.6)	3.3 ± 2.9
Spanish Water Dog	72	22 (30.6)	50 (69.4)	2.8 ± 2.5
Total	1 002	438 (43.8)	563 (56.2)	2.9 ± 2.7

Participant dogs took part in a test battery involving multiple tests, but not all dogs had results for every test included in this study (see Supplementary Table S1 for number of participant dogs in each test section). Most of the results for each test came from the same dogs, with some results missing, mostly because 17% of the dogs took part in a shortened version of the test battery (see Supplementary Materials S1 for details).

Most participant dogs were privately owned pet dogs. We did not have information on the training history of most dogs, apart from 31 police dogs (see Suppl. Table S2). A large proportion of the pet dogs were actively used in various dog sports (e.g., agility, scent work, obedience, etc.), based on discussions the testers have had with participating owners. Most dogs (including the police dogs) lived inside the house with their owners.

### Cognitive test battery

Dogs included in the study were participants in a commercial cognitive test battery (smartDOG™)^25^, which was developed by one of the authors (KT) based on previous scientific publications. Tests were performed by eight trained female smartDOG licence testers (including KT) at testing sites across Finland. We included 10 tests, seven of which measured cognitive traits and three of which measured behaviour. Descriptions of included tests are outlined in Table 2.

**Table 2:** Description of each test included in the study, in the order in which they were presented to the dog during the COGNITION test battery.

Test	Trait(s) measured	Short description
Greeting	Greeting behaviour towards an unfamiliar person	The dog’s behaviour upon first greeting the tester was rated on a scale of 1–7
Activity level	Activity level during the test	The dog was supplied with a FitBark^57^ accelerometer for the duration of the test battery, which provided an average activity level for the dog
Exploration	Dog’s exploration of a novel environment	The dog’s behaviour during its first few minutes in the testing environment was rated on a scale of 1–5
Cylinder test	Inhibitory control, impulsivity	Food is placed inside a transparent cylinder, and the dog has to inhibit their response to reach directly for the food, and instead go around the barrier to reach the food
Gestures	Social cognition, dog’s ability to understand human communicative cues	Object choice test: the human provides a gesture towards the bowl which contains food. Gestures included: dynamic distal pointing, momentary distal pointing, dynamic foot pointing, dynamic cross-forward pointing, and gaze
V-detour	Inhibitory control, spatial problem-solving ability	The dog is required to reach a food reward by detouring around a V-shaped fence
Unsolvable task	Social cognition, help-seeking behaviour, persistence, problem-solving strategy	The dog is faced with a problem where food is visible but out of reach. The dog’s response was measured as (a) human-directed behaviour, (b) independent behaviour, attempting to solve the task, and (c) abandoning the task; engaging in behaviours not directed at a human or the apparatus
Logical reasoning	Logical reasoning, ability to make inferences based on exclusion	The dog can see that one of two bowls is empty and has to infer that the treat is hidden under the other bowl
Memory vs gesture	Dog’s tendency to choose based on human gesture vs visual information, social cognition	The dog is required to choose between two bowls. The dog can see a human placing a treat in one bowl, and the human gestures towards the other (empty) bowl. The dog’s choice is then observed
Memory	Spatial short-term memory	The dog was required to remember the location of a food treat which was hidden under one of three bowls, for an increasing duration of time (from 1 to 2.5 min)

All tests involved solving various problems with food rewards. The owners were asked to bring the dog’s favourite treats, which were then used as rewards. In some cases, a toy was also used, if the dog was more motivated by toys than food. The owner was advised not to feed their dog prior to the test to ensure food motivation during the test. Fresh drinking water was available throughout the test. Testing took place indoors, and the minimum size of the testing room was approximately 30 m^2^. Most often only the tester, owner, and dog were present during testing, but occasionally family members were present as observers. The dog was off the lead throughout the test battery. A short break was included in the COGNITION test battery, during which the owner was asked to walk the dog outside for a maximum of two minutes.

The order of tests was the same for all dogs to ensure consistency across subjects. Each test included several trials. At the beginning of each trial, the tester always drew the dog’s attention, if necessary, by showing them a treat and saying the dog’s name. A (Finnish) word, such as “ok” or similar, was used to indicate to the owner the beginning of each trial. The owner was advised to release the dog upon hearing this word during the test battery. After each trial, the owner called or moved the dog back to the starting position using the collar and held the dog in place before releasing the dog for the next trial. The owner was advised to remain quiet and neutral when the dog was focusing on a task, but they were allowed to praise the dog when the dog ate a treat. Only during the V-detour was the owner allowed to encourage the dog to continue if the dog continuously stared at the owner. Between test sections, the dog was free to walk around for about 1–2 min.

A short description of each test is provided below, and more detailed information can be found in the Supplementary Information S1. An example of each cognitive test can also be found in the supplementary videos S1.

### Greeting

When the owner and dog first entered the test room, the dog’s response to the tester (an unknown person) was rated. The tester faced the dog while talking in a friendly voice and allowed the dog to approach herself. If the dog was not fearful or aggressive, the tester bent down and attempted to stroke the dog. The tester then continued stroking the dog as long as the dog was willing. This test lasted between 1–2 min in total.

The tester rated the dog’s response according to a scale ranging from 1 to 7. For analysis purposes, these scores were condensed into four groups: ‘fearful’ (score 1–3), ‘indifferent’ (score 4–5), ‘friendly’ (score 6), and ‘overexcited’ (score 7). The largest category (‘friendly’) was used as the reference category.

### Activity level

After the tester had greeted the dog, she attached a FitBark (FitBark Inc., Kansas City, MO, USA) activity monitor to the dog’s collar or harness. FitBark generates ‘BarkPoints’ (from here on referred to as ‘activity points’), which represent a proxy measure for the average activity level of the dog during the test battery. The monitor was kept on throughout testing and taken off when the test battery ended. Only dogs which had taken part in the COGNITION test battery were analysed.

### Exploration

After the FitBark had been attached, the dog was released and allowed to freely explore the test room for approximately five minutes. The tester rated the dog’s behaviour on a scale of 1–5. These scores were condensed into four groups: ‘low investigation’ (score 1–2), ‘moderate investigation’ (score 3), active investigation, walking (score 4), and very active investigation, running (score 5). The aim of this test was to measure the extent to which the dogs were willing to investigate a novel environment, which is thought to be an indication of curiosity, boldness, and activity level, whereas remaining by the owner’s side was thought to be a possible indication of fear, anxiety, or neophobia.

### The cylinder test

The cylinder test has been used extensively in animal cognition research to study impulsivity and inhibitory control^29^, more specifically the motor inhibitory response. Inhibitory control is a core executive function, which involves suppression of a prepotent but inefficient behaviour in favour of a more beneficial response. In this test, the dog is required to inhibit reaching directly for a visible food reward and instead detour around the transparent barrier to reach the reward.

The owner and dog were positioned 2–2.5 m away from the cylinder. During the training trials, the cylinder was opaque, and the dog was taught to access a food reward from either of the open sides. The experimenter stood directly behind the cylinder and placed a food reward inside while the dog was watching. The dog was then released and allowed to eat the treat (Supplementary Video S1). After the dog fulfilled the learning criteria (4 out of 5 trials without touching the outside of the cylinder), the test phase began. During the test phase the cylinder was transparent, and the dog was required to inhibit reaching for the now visible food directly, and to instead go around to the side of the cylinder to access the reward (Supplementary Video S2). Each trial during which the dog touched the outside of the cylinder was marked as an incorrect trial. If the dog ate the food without first touching the outside of the cylinder, this was marked as a correct trial. Percentage of correct responses (out of a total of 10 trials) was used as the response variable.

### Human gestures

Dogs’ understanding of pointing and other human gestures is often used as a measure of social cognition^30,31^. The test battery included five different gestures. Before the test phase, the dog was familiarized with the test set-up over four training trials, during which the dog was simply taught that food is available in either of the two bowls. (See Supplementary Video S3 for the training phase procedure.).

During the test phase, dogs took part in six trials for each gesture (30 trials in total). (See Supplementary Video S4 for the test procedure for each of the five gestures.) The percentage of correct responses (out of 30 trials) was calculated from all the gesture tests combined. The order of rewarded bowls was the same for each dog, starting with the left side. Every other trial was rewarded to the right and every other to the left. In order to ensure dogs were not learning this pattern, the percentage of correct responses from the final 6 trials (gaze) were compared to the percentage of correct responses from the first 6 trials (dynamic distal pointing) using a two-tailed paired t-test.

The trial always started with the dog and owner facing the tester, standing 2–2.5 m away. The tester showed a piece of food to the dog and placed it inside one of the bowls which she held in her hands. The bowls were placed on the floor in front of the tester, 95 cm apart from each other. Making sure the dog was watching, the tester provided the gesture (these are described in more detail below). After this, the dog was released and allowed to make a choice.

The procedure for each gesture was the same as described above. Each dog received the gestures in the same order (6 trials each): (1) dynamic distal pointing: the tester pointed at the correct bowl with an extended arm and index finger after which the dog was released, and the tester kept her arm in the same position while the dog made their choice, (2) momentary distal pointing: the tester pointed at the correct bowl with an extended arm and index finger for a duration of 2 s, after which the tester lowered her arm and the dog was released while the tester’s arms were flush at her sides, (3) dynamic proximal foot pointing: the tester placed the tip of her foot on the ground directly behind the correct bowl, and the dog was released while the tester remained in this position, (4) dynamic cross-forward pointing: THE tester used her contralateral arm to point at the correct bowl while rotating her shoulders in the same direction, sustaining this position while the dog made their choice, (5) gaze: the tester alternated her gaze between the dog and the bowl three times, and the dog was released while the tester maintained her gaze on the correct bowl.

### V-detour

The V-detour has been used in canine cognitive research to investigate spatial problem-solving ability^29^. The dog has to detour around a transparent V-shaped fence to access a food reward which is placed on the other side (Supplementary Video S5). Since the dog is required to move away from the visible treat to access it, the task is also often considered to measure inhibitory control.

The V-shaped fence was made out of compost fence panels, which were attached at an angle of approximately 70°. The owner and dog waited about 40 cm away from the intersecting angle of the V-shaped fence. The tester showed the dog several treats (or a toy) and placed them inside the fence while standing outside the fence. The owner released the dog while the dog was looking at the food. The number of seconds taken to solve the task was measured using a stopwatch. If the dog was not able to solve the task within 3 min, the trial was terminated.

### Unsolvable task

The unsolvable task has been used in canine cognitive research to assess persistence, problem-solving behaviour, human-directed communication, and social cognition^32^. In our version of this task, the dog was presented with four solvable trials, after which the task became impossible to solve (Supplementary Video S6).

The test involved a plastic or wooden box with a transparent lid, which had small holes to allow the dog to smell the food inside. During the four training trials, the dog was taught to access a treat placed inside the box by moving the plastic lid off. The difficulty of the trials increased gradually. Once the dog was successful in opening the lid, the test trial was begun. With the dog watching, the tester placed several treats inside the box. She then secured the lid in place so that it could not be opened, after which the owner released the dog. Both owner and experimenter remained quiet and still, looking only at the box during the subsequent 2-min period. The tester measured the time the dog spent on each behaviour: (a) independent problem-solving: attempting to solve the task independently, (b) human-directed behaviour: initiating social contact with either the tester or the owner, or (c) abandoning the task: not directing their behaviour toward the task or a person.

Three variables were used to measure the dogs’ behaviour during the unsolvable task. (1) Complete independence (comparing dogs which did not spend any time on human-directed behaviour to those which spent any amount of time on human-directed behaviour), (2) Percentage of time spent on human-directed behaviour, and (3) Abandoning task (comparing dogs which abandoned the task to those which never abandoned the task).

### Logical reasoning

This test aimed to measure the dog’s ability to make inferences based on exclusion^33^. The dog could see that one of two bowls was empty, and it had to infer that the treat was hidden under the other bowl. The tester sat on a chair or on the floor, about 1 m away from the dog. Two opaque bowls were placed upside down in front of the tester at arm’s length, one on the left and one on the right side. In each trial, a piece of food was placed under one of the bowls. The order of baiting was the same for each dog; first the left-hand bowl was baited, after which every other trial was baited to the right and every other trial to the left. The dog first took part in a training phase consisting of four trials, the aim of which was to familiarize the dog with the set-up and learn that there is always food hidden under one of the bowls. (See Supplementary Video S7 for the procedure of the training phase.).

When the dog correctly performed all four training trials, the test phase was initiated. This consisted of six trials. The tester held a writing pad as a visual barrier in front of the left-hand bowl and placed a treat under the bowl. She then placed the writing pad in front of the right-hand bowl and sham-baited the bowl. The writing pad was then removed, and the tester lifted the empty bowl up about 30 cm above the floor, keeping it there for about 1–2 s while the dog was watching. At the same time, the tester held her other hand on top of the baited bowl. The bowl was then placed back down, the tester placed her hands on her lap, and the dog was released. If the dog approached the correct bowl, they were allowed to eat the treat. The trial was then repeated another five times, every other trial rewarded on the right and every other on the left. (See Supplementary Video S8 for the procedure of the test phase.).

Two variables were used to measure logical reasoning of dogs: (a) percentage of correct responses and (b) understanding of the task. For percentage of correct responses, dogs were divided into three groups: (1) 0–50% of trials correct, (2) 51–82% of trials correct, and (3) 83–100% of trials correct. The tester also evaluated whether the dog had understood the task and was successfully making inferences based on exclusion. The dog was considered to have understood the task if at least 4 out of 6 trials were correct or the final 2–3 trials were correct.

### Memory vs gesture

Previous studies have shown that dogs are more likely to choose an empty bowl out of two choices if a human points towards it, even when they have seen that the other bowl has food in it^34–37^. Similar to the gesture tests, this test aims to measure social cognition. The procedure for this test was also similar to the gesture test, but instead of gesturing towards the baited bowl, the tester gestured towards the empty bowl. The tester used the gesture which the dog had been most successful with—this was often the dynamic distal point. The bowls remained on the floor throughout the test, and the treat was placed in one of the bowls while the dog was watching. The test consisted of two trials, with the first trial always baited to the left and the second trial baited to the right. (See Supplementary Video S9 for the procedure.) Dogs were divided into two groups: those which chose the baited container on both trials (relied on their memory), and those which chose the empty container on 1–2 trials (relied on the human’s gesture).

### Memory

The aim of this test was to measure the duration of the dogs’ short-term memory^38^. Three identical opaque bowls were placed upside down on the floor in a straight line, about 1 m apart from each other. A piece of food (or a toy) was placed under one of the bowls in each trial. The owner sat on a chair 3 m away from the middle bowl with the dog in front of her. During 7 training trials the dog learned to find a food reward from under one of the three bowls. Only a very short delay between hiding the food and releasing the dog was in place during this phase.

Once the dog had passed the training phase, the four test trials began. The tester placed a treat under one bowl while the dog was watching, after which a visual barrier was placed in front of the dog. The tester waited an increasing duration of time behind the owner and the dog (1st trial: 1 min, 2nd trial: 1.5 min, 3rd trial: 2 min, 4th trial: 2.5 min). After the waiting period, the tester removed the barrier, and the dog was released and allowed to make a choice. The order of baiting the bowls was the same for each dog: trial 1: middle, trial 2: left, trial 3: right, and trial 4: middle. (See Supplementary Video S11 for the procedure of the test phase.) Number of correct trials (out of a total of 4) was calculated for each dog. Since a very small number of dogs had a score of 0, these dogs were combined into a group with dogs that had a score of 1.

### Data analysis

All statistical analyses were performed using IBM SPSS Statistics Version 28. An alpha level of 0.05 was used for all statistical tests. Multiple and logistic regression analyses were used with the enter method to analyse differences between breeds for each variable. For ordinal variables, cumulative odds ordinal logistic regression with proportional odds was used. In each model, we included age and sex of the dogs as control variables, since previous research suggests these may affect various measures of cognition and behaviour^39–42^. Each model therefore included the predictors breed, age, and sex (apart from success in the V-detour, which only included breed as a predictor). The Labrador Retriever, one of the most popular breeds worldwide, was used as the reference breed, since it had the largest number of individuals tested out of all included pedigree breeds.

For the greeting variable, the data failed the assumption of proportional odds according to the full likelihood ratio test, and therefore a multinomial logistic regression was conducted instead of ordinal logistic regression. For success in the V-detour task, Fisher’s Exact test using the Monte Carlo method was run with 10,000 simulations, since an insufficient number of individuals failed to solve the task for a binary logistic regression to be reliable. For latency (s) to solve the V-detour task, the dependent variable was log-transformed to normalize its distribution. Since the cylinder test variable ‘percentage of correct responses’ was negatively skewed, a reflect and square root transformation was applied (i.e., each data point was subtracted from the maximum value plus 1, and a square root transformation was then applied to these scores). Due to the transformation, the variable was inverted, and therefore named ‘percentage of incorrect responses’ to aid with interpretability. For all transformed variables, the original values are included in the figures, whereas the transformed variables are reported in the tables and text.

### Ethical statement

We confirm that the procedures comply with national and EU legislation. Research was performed in accordance with the Declaration of Helsinki. The study was approved by the University of Helsinki Viikki Campus Research Ethics Committee (Statement 12/2021, accepted on 18/05/2021). Before participating in the cognitive test battery, each dog owner gave informed written consent for using their dogs’ test results in research. Reporting of results follows the recommendations of the ARRIVE guidelines. Informed consent was given by each subject for publication of identifying images/videos in an online-access publication.

## Results

### Greeting

A total of 934 dogs had results for the greeting test. Out of these, 12.8% were fearful when meeting the unknown person, 28.2% were indifferent, 41.1% responded in a friendly manner, and 11.1% of dogs had an overexcited greeting.

**Figure 1:**
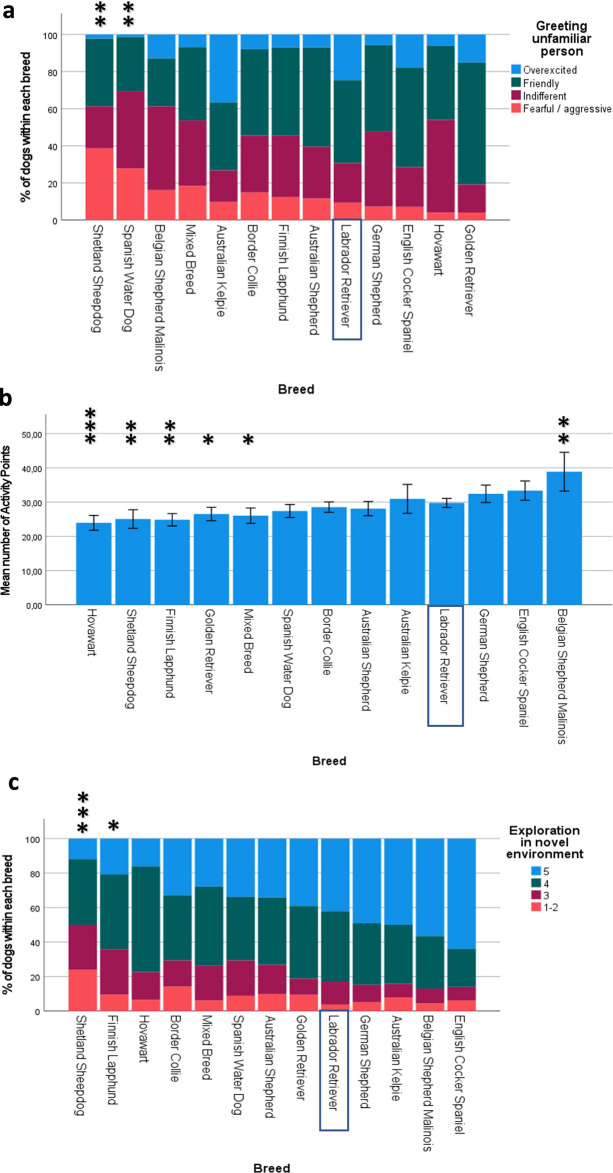
Breed differences of greeting unfamiliar person, activity level, and exploration in novel environment. Significant P-values (Bonferroni-corrected) are indicated with asterisks: ***p ≤ 0.001, **p ≤ 0.01, *p ≤ 0.05. The Labrador Retriever was used as the reference breed. (**a**) Percentage of dogs within each breed receiving each of the four greeting scores (n = 934). Breeds have been ordered based on odds ratios, from most fearful (highest odds ratio for score 1–3) on the left to least fearful (lowest odds ratio for score 1–3) on the right. P-values have been indicated for fearful response using asterisks. (**b**) Mean activity level scores (measured in FitBark activity points) for each breed (N = 759). Error bars represent 95% confidence intervals. Breeds are ordered based on B-values, with lowest activity levels on the left and highest activity levels on the right. (**c**) Percentage of dogs within each breed with each exploration score (N = 820). Breeds are ordered based on B-values, with lowest exploration on the left and highest exploration on the right. Score 1–2: low investigation (stays close to owner). Score 3: moderate investigation. Score 4: active investigation (walking). Score 5: very active investigation (running).

The multinomial regression model significantly predicted greeting scores over and above the intercept-only model (χ^2^(42) = 174.1, p < 0.001). The model explained 18.8% (Nagelkerke R^2^) of the variance in greeting behaviour. Sex did not have a significant effect on the prediction of greeting (χ^2^(3) = 2.65, p = 0.45), but age did (χ^2^(3) = 20.77, p < 0.001). An increase in age was associated with a decrease in the odds of having an overexcited greeting compared to a friendly greeting (which was used as the reference group) (χ^2^(1) = 9.94, p = 0.002). Breed had a significant effect on the prediction of greeting score (χ^2^(36) = 152.86, p < 0.001). See Fig. 1a for the proportions of greeting scores for each breed, and Supplementary Table S3 for parameter estimates for each greeting score.

### Activity level

A total of 759 dogs had activity level results available. Dogs received a mean of 29.4 FitBark activity points, ranging from 9.9 to 93.4 activity points. The multiple regression model significantly predicted activity levels of dogs (F (14) = 7.91, p < 0.001, adj. R^2^ = 0.12). There were 10 outliers but none of these were influential according to Cook’s Distance. Age and sex did not significantly predict activity level in dogs aged 1–8 years (age β = − 0.04, p = 0.23, sex β = − 0.03, p = 0.42). Six breeds differed significantly from the Labrador Retriever (which was used as the reference breed for all analyses). See Fig. 1b for mean activity points for each breed, and Supplementary Table S4 for parameter estimates.

### Exploration

A total of 820 dogs had results for exploration in a novel environment. Out of these, 8.3% showed low investigation, 15.8% showed moderate investigation, 39.7% showed active investigation (walking), and 36.3% showed very active investigation (running).

The ordinal regression model significantly predicted exploration scores over and above the intercept-only model (χ^2^(14) = 61.98, p < 0.001). The model explained 8% (Nagelkerke R^2^) of variance in exploratory behaviour. Age and sex did not have a significant effect on the prediction of exploration in dogs aged 1–8 years (age χ^2^(1) = 1.9, p = 0.17; sex χ^2^(1) = 0.05, p = 0.82), but breed did (χ^2^(12) = 57.68, p < 0.001). See Fig. 1c for percentage of each exploration score for each breed, and Supplementary Table S5 for parameter estimates.

### Cylinder test

**Figure 2:**
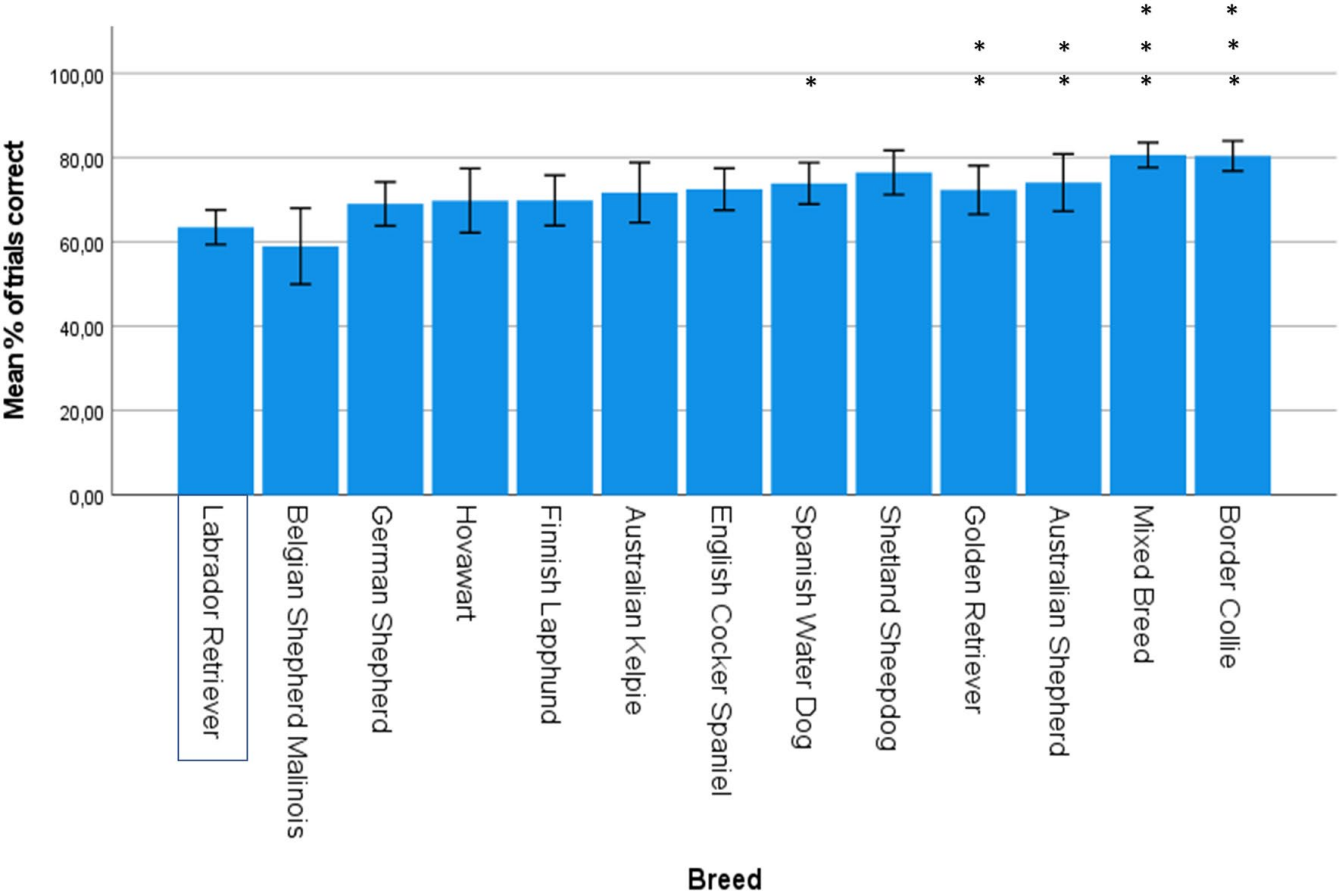
Mean percentage of *correct* trials for each breed in the cylinder test are presented, using the untransformed, original data (n = 992). Error bars represent 95% confidence intervals. Breeds are ordered based on B-values, with lowest success (low inhibitory control) on the left and highest success (high inhibitory control) on the right. The Labrador Retriever was used as the reference breed. Significant P-values (Bonferroni-corrected) are indicated with asterisks: ***p ≤ 0.001, **p ≤ 0.01, *p ≤ 0.05.

A total of 992 dogs took part in the cylinder test. The median success rate of dogs was 80% (IQR = 30%). The multiple regression model significantly predicted the percentage of incorrect trials in the cylinder test (F (14, 961) = 6.96, p < 0.001, adj. R^2^ = 0.08). No outliers were detected. Age was a significant predictor of performance in the cylinder test, β = 0.13, p < 0.001, with increasing age associated with an increasing percentage of incorrect trials (in dogs aged 1–8-years old). Sex was also a significant predictor of cylinder test performance, β = − 0.09, p = 0.004, with females making fewer mistakes than males. Five breeds had significantly lower percentages of incorrect trials compared to the Labrador Retriever. See Fig. 2 for the mean percentage of *correct* trials for each breed, and Supplementary Table S6 for parameter estimates.

### Human gestures

A total of 831 dogs took part in each of the five gesture tests. The mean percentage of correct trials for these dogs was 79% (SD = 12.9%). A two-tailed paired samples t-test showed that there was no significant change from the first 6 object choice trials to the last 6 trials, with a mean decrease of 0.84% in correct responses (95% CI [− 2.65, 0.96], t(831) = − 0.92, p = 0.36, d = -0.03). Therefore, it is unlikely that dogs were learning the pattern for baiting the bowls.

**Figure 3:**
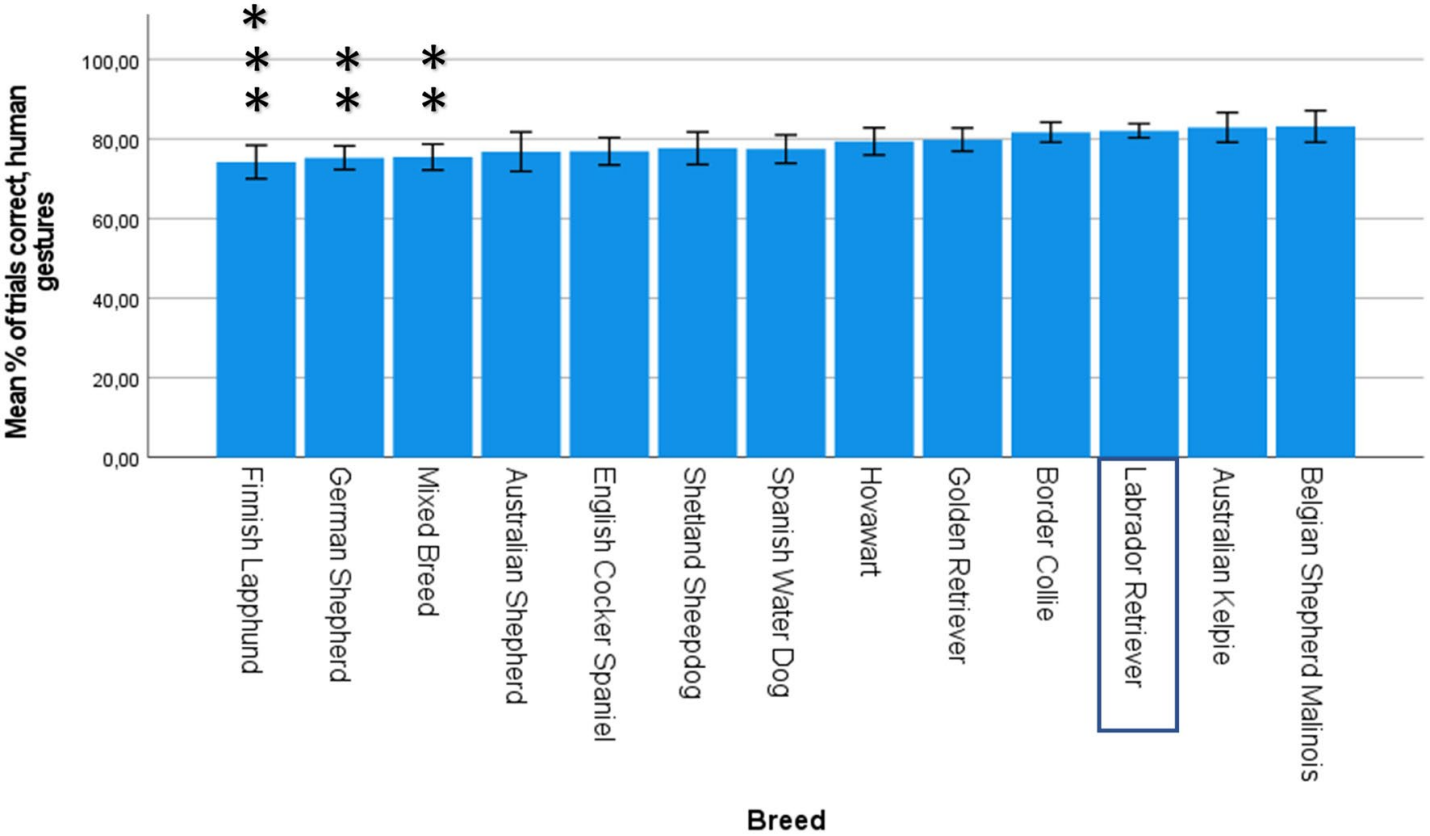
Mean percentage of correct trials in the gesture tests within each breed (n = 831). Error bars represent 95% confidence intervals. Breeds have been ordered based on B-values, with lowest success on the left and highest success on the right. The Labrador Retriever was used as the reference breed. Significant P-values (Bonferroni-corrected) are indicated with asterisks: ***p ≤ 0.001, **p ≤ 0.01, *p ≤ 0.05.

The multiple regression model significantly predicted the percentage of correct trials for the gesture tests (F (14, 804) = 3.41, p < 0.001, adj. R^2^ = 0.04). There were two outliers, but these were not influential according to Cook’s Distance. Age and sex did not significantly predict performance in dogs aged 1–8 years (age β = 0.03, p = 0.45; sex β = − 0.06, p = 0.12). Three breeds had significantly lower scores than the Labrador Retriever. See Fig. 3 for the mean percentage of correct trials for each breed, and Supplementary Table S7 for parameter estimates.

### V-detour

**Figure 4:**
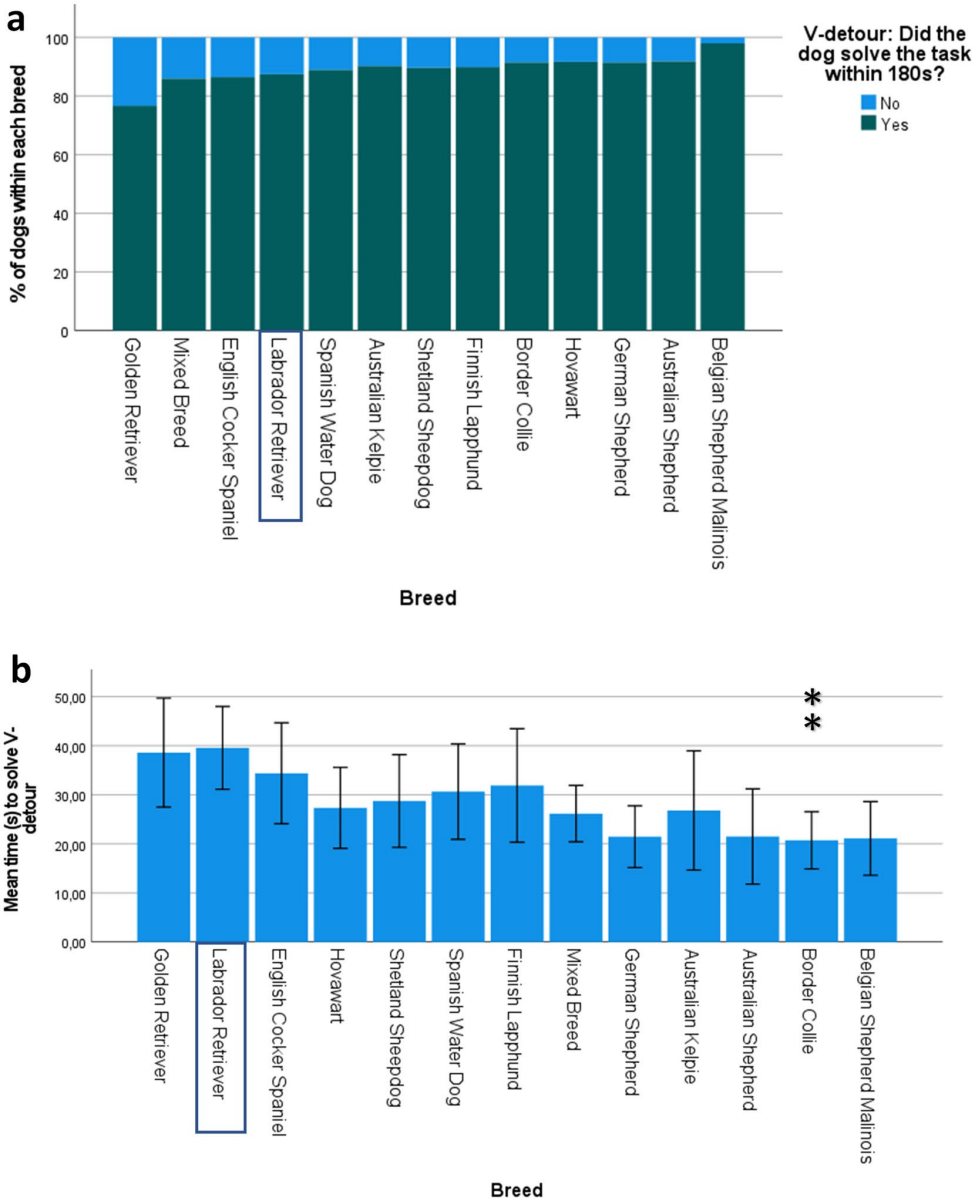
Performance of dogs during the V-detour task. The Labrador Retriever was used as the reference breed. Significant P-values (Bonferroni-corrected) are indicated with asterisks: ***p ≤ 0.001, **p ≤ 0.01, *p ≤ 0.05. (**a**) Percentage of dogs within each breed succeeding vs failing in the V-detour task (n = 993). Breeds have been ordered based on B-values, with lowest succeeding breeds on the left and highest succeeding breeds on the right. (**b**) Mean latency (s) to solve the V-detour for each breed are presented using the untransformed, original data (n = 863). Error bars represent 95% confidence intervals. Breeds are ordered based on B-values, with lowest succeeding breeds (took a long time to solve the task) on the left and highest succeeding breeds (solved the task quickly) on the right.

A total of 993 dogs took part in the V-detour test. Out of these, 88.5% successfully solved the V-detour within 3 min. There was no significant difference in the proportions of dogs within each breed succeeding compared to those failing (p = 0.13, 99% CI [0.12, 0.14]). See Fig. 4a for the proportion of successful dogs in each breed.

The median time for solving the V-detour task (when unsuccessful dogs were removed from the analysis) was 12 s (IQR = 29 s). The multiple regression model significantly predicted the time taken to solve the task (n = 863, F (14, 848) = 2.52, p = 0.002, adj. R^2^ = 0.02). No outliers were detected. Age and sex did not significantly predict performance in the V-detour in dogs aged 1–8 years (age β = 0.05, p = 0.14; sex β = − 0.05, p = 0.12). The Border Collie had a significantly lower predicted mean log score compared to the Labrador Retriever (β = − 0.14, p = 0.01). See Fig. 4b for mean time taken to solve the task for each breed, and Supplementary Table S8 for parameter estimates.

### Unsolvable task

**Figure 5:**
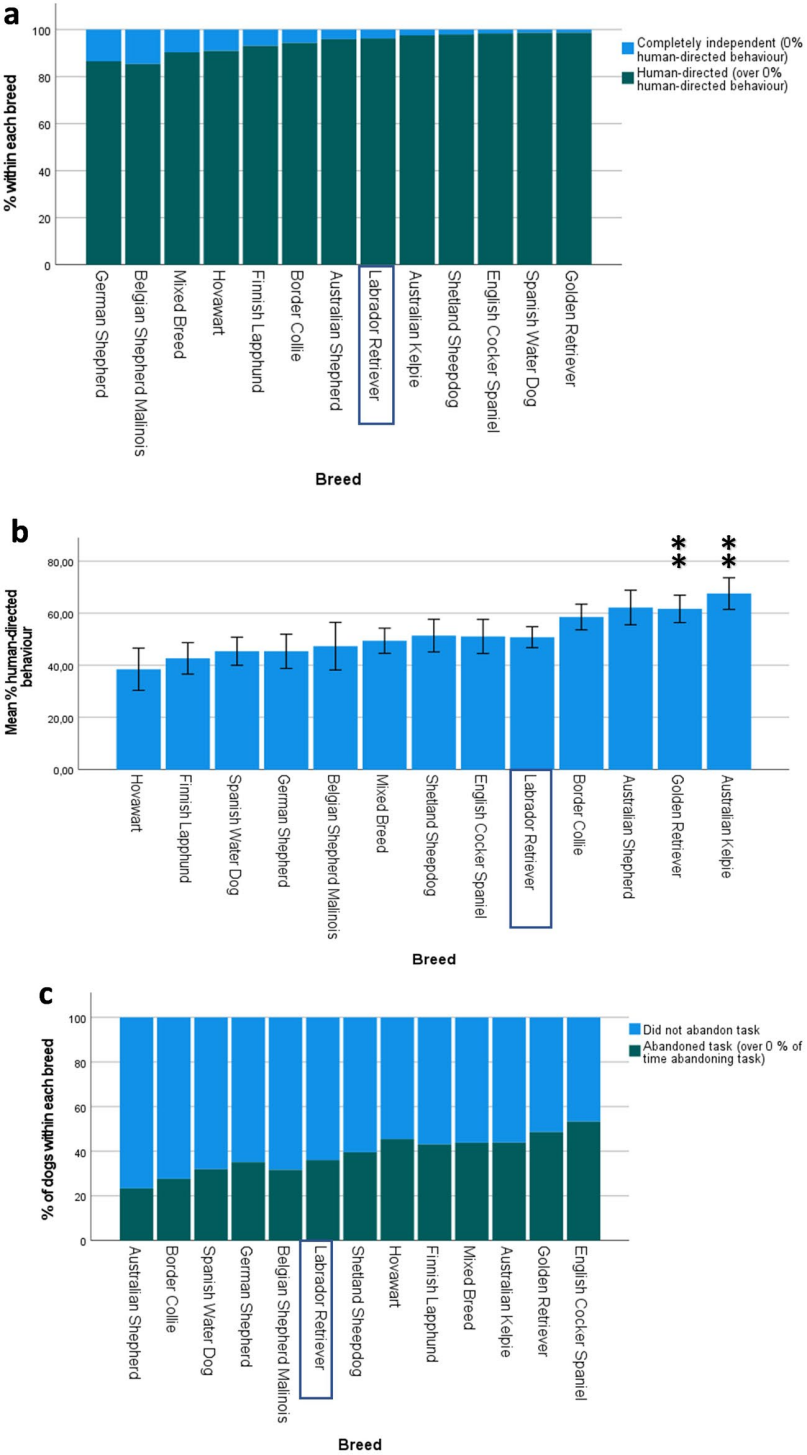
Performance of breeds in the unsolvable task (n = 969). The Labrador Retriever was used as the reference breed. Significant P-values (Bonferroni-corrected) are indicated with asterisks: ***p ≤ 0.001, **p ≤ 0.01, *p ≤ 0.05. (**a**) Percentage of dogs within each breed belonging to the human-directed compared to the completely independent groups during the Unsolvable Task. Breeds have been ordered based on odds ratios, with most independent breeds (most likely to spend 0% of their time on human-directed behaviour) on the left and least independent breeds (least likely to spend 0% of their time on human-directed behaviour) on the right. (**b**) Mean percentage of time (out of a total of 120 s) spent on human-directed behaviour during the unsolvable task for each breed. Error bars represent 95% confidence intervals. Breeds are ordered based on B-values, with lowest amount of time spent on human-directed behaviour on the left and highest amount of time on the right. (**c**) Percentage of dogs within each breed abandoning the task (over 0% of time spent on abandoning task) vs persisting (0% of time spent on abandoning task) with the unsolvable task. Breeds are ordered based on odds ratios, with the most persistent breeds (least likely to abandon task) on the left and the least persistent breeds (most likely to abandon task) on the right.

A total of 969 dogs took part in the unsolvable task. Only 57 (5.7%) of these were completely independent during the unsolvable task (i.e., they spent 0% of their time on human-directed behaviour). When comparing these dogs to those which spent over 0% of their time on human-directed behaviour, the binomial logistic regression model was statistically significant (χ^2^(14) = 34.28, p = 0.002). The model explained 9.7% (Nagelkerke R^2^) of the variance. Age was not a significant predictor of independence in dogs aged 1–8 years (χ^2^(1) = 0.59, p = 0.44), but females had lower odds of being completely independent compared to males (χ^2^(1) = 5.22, p = 0.02). Breed was also a significant predictor of independence (χ^2^(12) = 23.13, p = 0.03), but no breed was significantly different from the Labrador Retriever. See Fig. 5a for the percentage of completely independent dogs within each breed, and Supplementary Table S9 for parameter estimates.

When completely independent dogs had been removed from the analysis, the median amount of time spent on human-directed behaviour was 54.2% (65 s out of a total of 120 s) (IQR = 40%). The multiple regression model significantly predicted the percentage of time spent on human-directed behaviour (n = 912, F (14, 885) = 5.7, p < 0.001, adj. R^2^ = 0.07). No outliers were detected. Age significantly predicted time spent on human-directed behaviour (β = 0.09, p = 0.009), with older dogs spending a larger proportion of their time on human-directed behaviour. Sex did not significantly predict time spent on human-directed behaviour (β = 0.002, p = 0.95). The Golden Retriever and Australian Kelpie had significantly higher scores than the Labrador Retriever. See Fig. 5b for the percentage of time spent on human-directed behaviour for each breed, and Supplementary Table S10 for parameter estimates.

A total of 37.2% of dogs spent over 0% of their time abandoning the task. When comparing these dogs to those which persisted with the task (0% of time spent on abandoning the task), the binomial logistic regression model was statistically significant (χ^2^(14) = 40.89, p < 0.001). The model explained 5.7% (Nagelkerke R^2^) of the variance. Age was a significant predictor (χ^2^(1) = 16.34, p < 0.001), with older dogs being less likely to abandon the task. Breed was a significant predictor of abandoning the task, χ^2^(12) = 24.16, p = 0.02, but sex was not (χ^2^(1) = 0.32, p = 0.57). No breed significantly differed from the Labrador Retriever. See Fig. 5c for the percentage of dogs within each breed abandoning the task, and Supplementary Table S11 for parameter estimates.

### Logical reasoning

A total of 826 dogs took part in the logical reasoning task. Out of these, 33.4% were rated as having understood the logical reasoning task. When comparing dogs which understood the task to dogs which failed to understand the task, the binomial logistic regression model was not statistically significant (χ^2^(14) = 20.95, p = 0.1). See Supplementary Fig. S5a for the percentage of dogs within each breed which understood the task.

For percentage of correct trials, 60.9% of dogs were correct on 0–50% of trials, 22.6% of dogs were correct on 51–82% of trials, and 16.5% of dogs were correct on 83–100% of trials. The ordinal regression model did not significantly predict success in the logical reasoning task above the intercept-only model, χ^2^(14) = 20.03, p = 0.13. See Supplementary Fig. S5b for the percentage of dogs within each breed with different rates of success.

### Gesture vs memory

**Figure 6:**
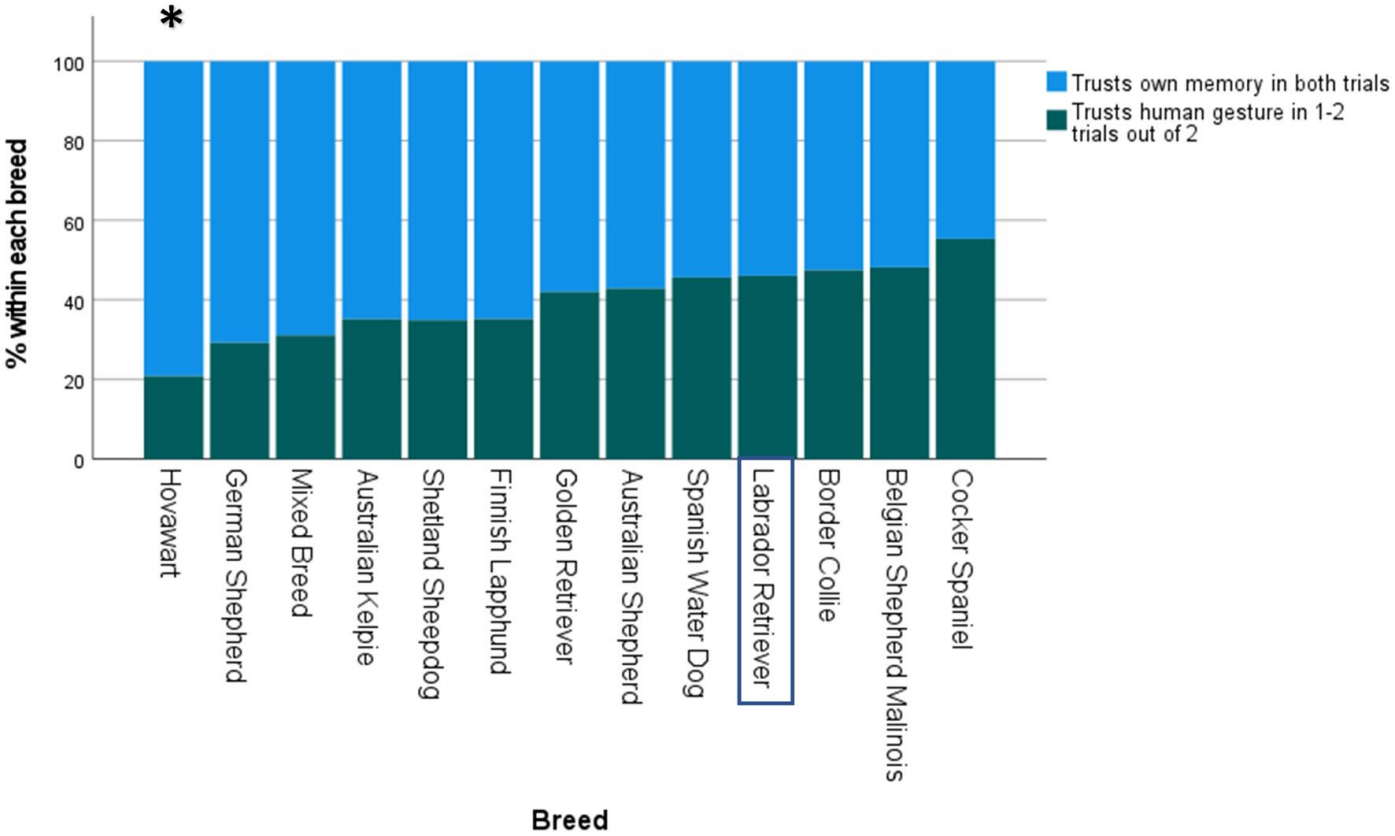
Percentage of dogs within each breed which trusted the human gesture compared to those which trusted their own memory (n = 823). Breeds have been ordered based on odds ratios, with breeds which are most likely to trust their own memory on the left and breeds which are most likely to trust the human’s gesture on the right. Significant P-values (Bonferroni-corrected) are indicated with asterisks: ***p ≤ 0.001, **p ≤ 0.01, *p ≤ 0.05. The Labrador Retriever was used as the reference breed.

A total of 823 dogs took part in the gesture vs memory test. Out of these, 40.5% followed the human gesture to the baited container in at least one trial. The binomial logistic regression model for trusting the human gesture was statistically significant (χ^2^(14) = 29.23, p = 0.01). The model explained 4.8% (Nagelkerke R^2^) of the variance in the gesture vs memory test. Age and sex were not significant predictors in dogs aged 1–8 years, but breed was a significant predictor of trusting the gesture, χ^2^(12) = 25.42, p = 0.01. See Fig. 6 for percentage of dogs within each breed which trusted the human gesture vs their own memory, and Supplementary Table S12 for parameter estimates.

### Memory

A total of 822 dogs took part in the memory test. Out of these, 14.1% were correct on 0–1 trials, 30.4% were correct on two out of four trials, 35.3% were correct on three trials, and 20.2% were correct on all four trials. The ordinal logistic regression model did not significantly predict the number of correct trials in the spatial memory task above the intercept-only model (χ^2^(14) = 19.26, p = 0.16). See Supplementary Fig. S6 for the percentage of dogs within each breed with different success rates.

## Discussion

We found significant differences between individual dog breeds for five of the seven cognitive tests included in the test battery. Breed differences were found for measures of social cognition, persistence, inhibitory control, and spatial problem-solving ability. Differences were also evident for activity level, greeting of an unfamiliar person, and exploration of a novel environment. In contrast, no breed differences were found for short-term memory or logical reasoning.

Both inhibitory control and social cognition are likely to be especially important traits during artificial selection of dog breeds, both historically and in the present day. For example, inhibitory control may be a valued trait in herding dogs, which are required to inhibit their predatory responses. The Border Collie and Australian Shepherd were among the highest-scoring breeds in the cylinder test, indicating high inhibitory control. In contrast, the Malinois and German Shepherd were some of the lowest-scoring breeds. These breeds are often used in working roles requiring high responsiveness, which is often associated with low inhibitory control and high impulsivity^43,44^. Human-directed behaviour and socio-cognitive abilities may be highly valued in pet dogs and breeds required to work closely with people, such as herding dogs and retrievers^45,46^. In line with this, the Kelpie, Golden Retriever, Australian Shepherd, and Border Collie spent the largest proportion of their time on human-directed behaviour during the unsolvable task. In contrast, the ability to work independently may be important for various working dogs, such as detection dogs^47,48^. In our study, the two breeds which were most likely to be completely independent during the unsolvable task (spending 0% of their time on human-directed behaviour) were the German Shepherd and Malinois.

In conclusion, many of our results seem to reflect the breeds’ original or current function, but several breed differences could not be easily explained by breed function alone. For example, many of the breeds in our study belonged to the herding group, but there was often large variation in their results in several cognitive tests. The Finnish Lapphund received the lowest score in the gesture tests, whereas the Kelpie and Malinois had the highest scores for this test, despite all three being herding breeds. Similarly, during the unsolvable task, the Australian Shepherd was the least likely breed to abandon the task, whereas the Kelpie was among the breeds most likely to abandon the task. It seems evident that breeds can vary behaviourally from each other even within their breed groups, since different traits may have been (both intentionally and unintentionally) selected for in different breeds, despite the breed group they belong to.

Our results replicate the findings of various studies which have investigated differences between individual breeds in the V-detour task^19^, understanding of human gestures^7,8^, and the unsolvable task^8,18^. In contrast, most studies which have failed to find breed differences have compared breed groups^10,11,13,19,49,50^. It is possible that potential differences between breeds may have been missed in these studies, since (as shown by our current study) behavioural variation within breed groups can be substantial. For example, the Golden Retriever differed significantly from the Labrador Retriever in the unsolvable task and the gesture test, even though both belong to the retriever group. Our findings therefore highlight the importance of investigating behavioural differences between individual breeds rather than only relying on breed group categorizations.

We found no significant breed differences for logical reasoning or memory, and these results seem to be in line with previous studies^12,13^. However, it is important to note that even though we did not find differences between these specific breeds, this does not mean they do not exist between other breeds. For example, we did not include ancient breeds, hounds, or terriers in our analysis. Therefore, more research on individual breed differences on these cognitive traits is warranted.

It is important to note that the population used in this study is not representative of the entire dog population, since findings may differ across countries and cultures. In addition, since we used a commercial test battery, only certain types of owners and dogs were inevitably self-selected. The breeds included in our study were mostly breeds used in dog sports, and most owners were active in various dog sports or competitions. Even though this limits the generalization of our results, it is noteworthy that significant breed differences emerged despite the similarity of participant dogs and their training histories.

There is a possibility that the differences seen in our study were not based on genetic differences between breeds but rather due to variation in life experiences or training, since these have also been found to influence behaviour in cognitive tests^8,13,49,51,52^. Unfortunately, we were not able to control for the possible effects of training, environment, life experiences, or background of the dogs, since this information was not available to us. Therefore, the extent of their possible effects on breed differences in our study is not known; however, this is something we will investigate next. Previous studies suggest that environmental effects are unlikely to be the only explanation for breed differences. Genetic relatedness between dog breeds has been shown to account for a substantial portion of variation in understanding human gestures and inhibitory control^9^. In addition, when training history of participating dogs has been controlled for, significant breed differences have still been found in the unsolvable task^18,27,53^, problem-solving tasks^20,49^, inhibitory control^9^, and point-following ability^9,54^. However, this topic warrants further investigation to determine the extent of heritable differences between breeds as opposed to the effect of life experiences.

It could be argued that the large proportion of police dogs within the Malinois breed (45%) could have biased breed differences in our study. However, our sample also consisted of other working dogs as well as several pet dogs which participated extensively in training and competitions, but we did not have information about these individuals. Therefore, it would not have made much sense to exclude police dogs. We nevertheless analysed the cylinder test, V-detour, and unsolvable task (which were the only tests which police dogs took part in) with police dogs excluded. As a result, breed differences in the proportions of completely independent dogs in the unsolvable task became non-significant, and the proportion of completely independent Malinois dropped from 14.6 to 7.7%. For all other variables breed differences were still significant and the scores of Malinois changed only slightly (results not shown). Therefore, it seems that the inclusion of police dogs only affected complete independence during the unsolvable task but did not influence other results to a great extent.

Another factor which could affect breed differences is the brain size and skull shape of the breeds^13,54–56^. However, the breeds included in our study all had similar skull shapes, and there were no extremes in body size. The Shetland Sheepdog and Cocker Spaniel were the only breeds which markedly differed in size from the others, but they did not seem to consistently differ from the larger breeds in their results.

In conclusion, we found significant breed differences for various behavioural and cognitive traits in dogs. This is one of the few studies investigating individual breed differences in dogs, especially in non-social cognitive traits which are rarely studied in this context. Our results provide a more complete picture of breed-typical behaviour in dogs.

## Supplementary Information

Supplementary Video 1.

Supplementary Video 2.

Supplementary Video 3.

Supplementary Video 4.

Supplementary Video 5.

Supplementary Video 6.

Supplementary Video 7.

Supplementary Video 8.

Supplementary Video 9.

Supplementary Video 10.

Supplementary Video 11.

Supplementary Information 1.

## Data Availability

The datasets analysed during the current study are not publicly available due to privacy restrictions but are available from the corresponding author on reasonable request.
