# Supplementary material for: Breed differences in social cognition, inhibitory control, and spatial problem-solving ability in the domestic dog (Canis familiaris)
Source: Sci Rep. 2022 Dec 29;12:22529. doi: 10.1038/s41598-022-26991-5 (PMC9800387; doi:10.1038/s41598-022-26991-5)
Supplement: Supplementary file 12 — Supplementary Information 1. [file 41598_2022_26991_MOESM12_ESM.docx]

**Supplementary Information**

**Methods**

**Test Battery**

Most of the dogs (n = 827, 82.6 %) took part in a test battery called COGNITION. This lasted approximately 1.5 hours and included 11 tests which measured cognitive and behavioural traits of dogs. All these tests were included in our analysis apart from a test measuring social learning (this was performed last in the COGNITION test battery). The remaining 175 (17 %) dogs took part in a shorter test battery which included 3―7 tests. The tests included in each of the short test batteries are outlined below:

81 dogs: greeting, exploration, cylinder test, dynamic distal pointing, V-detour, unsolvable task

40 dogs: greeting, activity level, exploration, cylinder test, dynamic distal pointing, V-detour, unsolvable task

31 dogs (police dogs): activity level, cylinder test, V-detour, unsolvable task

17 dogs: greeting, activity level, exploration, cylinder test, V-detour, unsolvable task

5 dogs: greeting, gesture tests, unsolvable task

1 dog: greeting, gesture tests, unsolvable task, memory

In addition, some dogs had missing data due to not passing the required criteria for the training phases, not being motivated by the reward, faulty testing equipment, being too afraid of the test situation, breaking the test apparatus, or being already familiar with the testing environment (in which case the exploration score was not included). Tests for greeting and exploration were not added to the test batteries until August 2016, which is why results for these tests are missing from dogs tested before this time. Supplementary Table 1 shows the total number of dogs from each breed included in each test.

| Breed | Greeting | Activity level | Explora-tion | Cylinder Test | Gestures | V-detour | Unsol-vable Task | Logical Reasoning | Gesture vs Memory | Memory |
| --- | --- | --- | --- | --- | --- | --- | --- | --- | --- | --- |
| Australian Shepherd (n = 49) | 43 | 41 | 41 | 49 | 44 | 49 | 49 | 42 | 42 | 42 |
| Belgian Shepherd Malinois (n = 49) | 31 | 24 | 23 | 49 | 27 | 49 | 41 | 29 | 27 | 26 |
| Border Collie  (n = 106) | 101 | 96 | 85 | 105 | 101 | 105 | 105 | 100 | 99 | 101 |
| English Cocker Spaniel (n = 60) | 56 | 48 | 50 | 60 | 56 | 59 | 60 | 57 | 56 | 56 |
| Golden Retriever (n = 74) | 73 | 56 | 64 | 73 | 67 | 73 | 74 | 69 | 69 | 68 |
| Australian Kelpie (n = 41) | 41 | 33 | 38 | 41 | 36 | 41 | 41 | 37 | 37 | 37 |
| German Shepherd (n = 82) | 69 | 62 | 59 | 82 | 65 | 82 | 74 | 65 | 65 | 65 |
| Mixed Breed (n = 149) | 147 | 68 | 133 | 149 | 76 | 149 | 145 | 75 | 74 | 74 |
| Shetland Sheepdog (n = 48) | 44 | 42 | 42 | 48 | 44 | 48 | 48 | 42 | 43 | 43 |
| Finnish Lapphund (n = 59) | 57 | 50 | 53 | 59 | 54 | 59 | 58 | 53 | 54 | 54 |
| Hovawart (n = 50) | 50 | 48 | 31 | 47 | 50 | 48 | 44 | 48 | 48 | 48 |
| Spanish Water Dog (n = 72) | 72 | 52 | 68 | 72 | 57 | 72 | 72 | 56 | 57 | 56 |
| Labrador Retriever (n = 163) | 150 | 139 | 133 | 158 | 154 | 159 | 158 | 153 | 152 | 152 |
| Total | **934** | **759** | **820** | **992** | **831** | **993** | **969** | **826** | **823** | **822** |

**Supplementary Table 1.** Number of dogs (out of the total of 1,002 participant dogs) with test results for each test section, divided by breed.

Six dogs took part in two different test batteries at different time points as adults; if these dogs had missing results for some tests, their results from different test sections were combined. If the dog had completed the same test two different times, only the results from their first test battery were included in the analysis. In addition, 28 dogs had previously taken part in a test battery designed for puppies (see Supplementary Table 2 for breeds), which was a shorter version of the adult test battery. For these dogs, only the results from their adult test battery were included. The puppy test battery included the tests activity level, greeting, exploration, cylinder test, gesture tests (excluding momentary distal pointing and pointing with the foot), V-detour, and unsolvable task. These tests were performed in the same order as in the COGNITION test battery.

| Breed | Took part in a puppy test between the ages of 3―8 months | Confirmed police dogs |
| --- | --- | --- |
| Australian Shepherd | 1 |  |
| Belgian Shepherd Malinois |  | 22 |
| Border Collie | 1 |  |
| English Cocker Spaniel | 4 |  |
| German Shepherd | 2 | 7 |
| Golden Retriever | 6 |  |
| Labrador Retriever | 10 | 2 |
| Mixed Breed | 2 |  |
| Shetland Sheepdog | 1 |  |
| Total | 28 | 31 |

**Supplementary Table 2.** Number of dogs from each breed which participated in a puppy test in addition to the adult test battery and number of dogs in each breed which are confirmed police dogs. This is not a definitive list of working dogs, since there may have been other working dogs among tested dogs.

The test sections included in our analysis are described in detail below, in the order in which they appear in the COGNITION test battery.

**Greeting**

When the owner and dog entered the test room, the dog still on a lead, the dog’s first response to the tester (an unknown person) was rated. This was based on a previous study^1^, in which dogs’ results in a similar greeting test strongly correlated with the owners’ evaluation of the dogs’ fear towards strangers. Greeting was done in a natural way; when the dog entered the room, the tester faced the dog while talking in a friendly voice and allowed the dog to approach herself. If the dog showed signs of fear (most often avoidance or increasing distance from the tester) or aggression, the tester did not try to make contact with the dog. Otherwise, the tester approached the dog, bent down, and attempted to stroke the dog. The tester then continued stroking the dog as long as the dog was willing. This test lasted between 1―2 minutes in total.

The tester rated the dog’s response according to the following scale:

7 = Over-excited greeting, jumps up or barks

6 = Initiates contact with person or reciprocates contact initiated by person

5 = Accepts contact initiated by person

4 = Somewhat indifferent towards person, interested in other things

3 = Passive, careful, stays still, but has the courage to greet person

2 = Avoids contact, backs away, or pulls tail down

1 = Rejects contact by trying to bite / growling / barking

Due to the small number of individuals with scores of 1―5, scores of 1―3 were combined into one group and scores of 4―5 into another group. This resulted in four groups of dogs:

1. Fearful (score 1―3),
2. Indifferent (score 4―5),
3. Friendly (score 6),
4. Overexcited (score 7)

The largest category (‘friendly’) was used as the reference category.

**Activity Level**

After the tester had greeted the dog, she attached a FitBark (FitBark Inc., Kansas City, MO, USA)^2^ activity monitor to the dog’s collar or harness. If the dog was fearful or aggressive, the owner was asked to attach the device instead. FitBark is a 3D accelerometer which has been shown to be a valid tool for measuring a dog’s off lead activity levels^3^. The tracker measures the dog’s physical activity level several times per second, and readings are then integrated over a 1-minute epoch. Based on the average of these readings, FitBark generates ‘BarkPoints’, which represent a proxy measure for the average activity level of the dog during the test battery (from here on referred to as ‘activity points’). The monitor was kept on throughout testing and taken off when the test battery ended. Only dogs which had taken part in the COGNITION test battery were analysed, in order to make sure that participants had the same test protocol and differences in activity levels were not due to differences in the length of the test battery or the test sections included.

**Exploration**

After the FitBark had been attached, the owner was advised to release the dog. The dog was then allowed to freely explore the test room while the owner filled in an information sheet. The dog’s behaviour in the novel environment was rated during approximately five minutes according to the following scale:

5 = The dog is very active, runs and investigates the whole room

4 = The dog is active, walks mostly, investigates the whole room

3 = The dog is calm, walks and investigates to some extent

2 = The dog investigates to some extent but mostly remains by the owner’s side

1 = The dog remains by the owner’s side the whole time

This test is similar to an open-field test^4^ which is a widely used procedure for measuring activity, curiosity, and willingness to explore in animals. It is also thought to measure emotionality, fear, neuroticism, anxiety, shyness, and neophobia at one end of the scale, and exploratory behaviour, boldness, neophilia, and extroversion at the other end of the scale^5-11^. Exploration in an unfamiliar environment has also been used to investigate the dog’s level of attachment to its owner, with securely attached dogs engaging in more independent exploration in the presence of the owner, whereas dogs with an insecure-ambivalent attachment style remain by the owner’s side^12,13^.

Due to small numbers of dogs with scores of 1 and 2, these were combined into one group. This resulted in four groups of dogs:

1. Low investigation (score 1-2)
2. Moderate investigation (score 3)
3. Active investigation, walking (score 4)
4. Very active investigation, running (score 5)

**The Cylinder Test**

The cylinder test has been used extensively in animal cognition research to study impulsivity and inhibitory control^14^, specifically the motor inhibitory response. In this test, the dog is required to inhibit reaching directly for a visible food reward and to instead go around the transparent barrier to reach the reward. Each trial during which the dog touches the outside of the cylinder is marked as an incorrect trial.

The owner and dog were positioned 2―2.5 m away from the plastic cylinder (20 x 25 cm), which was attached to a wooden board placed on the floor. The tester held her foot on top of the wooden board to prevent the cylinder from moving around. The cylinder was placed so that the open sides did not face toward the dog. During the training trials, the cylinder was opaque, so that the dog could not see the treat inside until moving to the side, and the dog was taught to access a food reward from either of the open sides. The experimenter stood directly behind the cylinder (so that the cylinder was between the dog and the tester), showed the dog a food reward and placed it inside the cylinder while the dog was watching. If the dog was not looking in the direction of the cylinder, the tester said the dog’s name to get their attention. After the treat was placed inside the cylinder, the tester stood directly behind the cylinder with arms flush at her sides. The dog was then released and allowed to eat the treat, after which the owner collected the dog back to the starting position, and the trial was repeated. (See Supplementary Video 1 for the procedure of the training phase) After the dog fulfilled the learning criteria (4 out of 5 trials without touching the outside of the cylinder), the test phase began.

During the test phase, the cylinder was made transparent (by taking out a black cylinder made of cardboard from inside the transparent plastic cylinder). The dog was required to inhibit reaching for the now visible food directly, and to instead go around to one of the sides of the cylinder to access the reward (Supplementary Fig. 1, Supplementary Video 2). Apart from the treat now being visible through the transparent cylinder, the procedure was otherwise identical to the training trials; the tester placed a piece of food inside the cylinder while the dog was watching, and the dog was released. During the trial, both the owner and tester remained stationary with arms flush at their sides, without talking to the dog or helping in any way. If the dog ate the food without first touching the outside of the cylinder with nose or paw, this was marked as a correct trial. If the dog touched the outside of the cylinder one or more times, this was marked as an incorrect trial. The dog was always allowed to eat the reward whether the trial was correct or incorrect. After each trial, the owner collected the dog back to the starting position and the trial was repeated. Percentage of correct responses (out of a total of 10 trials) was used as the response variable.


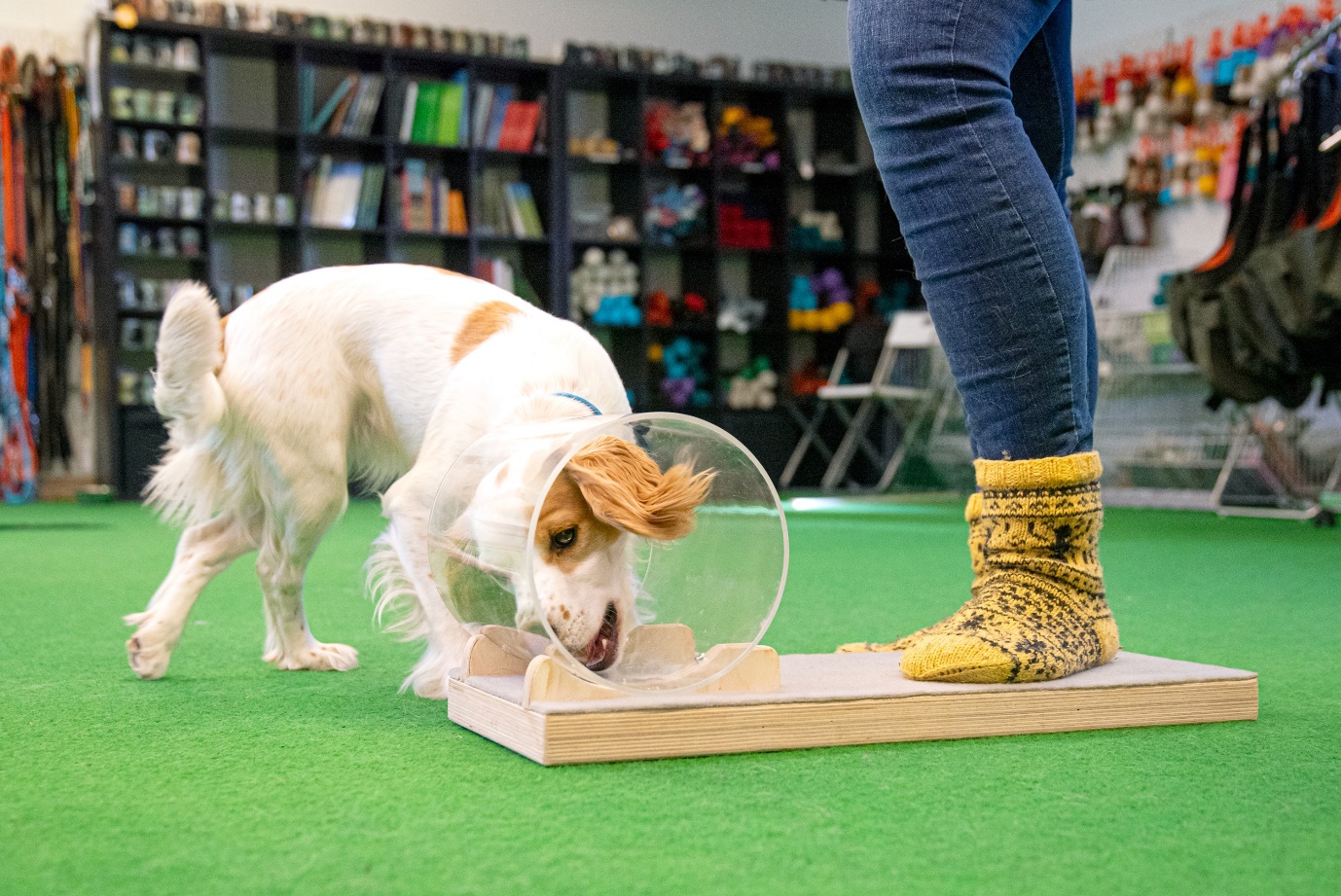


**Supplementary Figure 1**. Test phase of the Cylinder Task. The dog has succeeded in reaching the food reward inside the transparent cylinder. Image by Mainossatama Oy.

**Human Gestures**

Dogs are able to follow human gestures, such as pointing with a hand, from a very early age^16,17^. The test battery included five object choice tasks with five different gestures: 1) dynamic distal pointing, 2) momentary distal pointing, 3) dynamic foot pointing, 4) dynamic cross-forward pointing, and 5) gaze. These have all been extensively researched to understand the social cognition of dogs^18,19^. Multiple studies have shown that dogs in this situation do not generally use olfactory cues to find the food, since in control trials dogs do not perform better than would be expected by chance^20-25^. Only dogs which had participated in each of the five gesture tests were included in the analysis.

***Training Phase***

Before the test phase, the dog was familiarized with the test set-up during four trials. The owner held the dog 2―2.5 metres away, facing the tester who stood facing the dog. The tester placed an opaque, white plastic bowl (diameter 17 cm, height 13 cm) on the floor, about 50 cm to the left side of her. The bowl was high enough so that the dog could not see inside without getting close. To get the dog’s attention, the tester showed the dog a piece of food and (if the dog was not looking in the direction of the tester) said the dog’s name. The tester then placed a piece of food inside the bowl while the dog was watching. The tester then returned to a neutral standing position, and the dog was released and allowed to eat the food from the bowl. During this time, the tester and the owner remained stationary and quiet. If the dog refused to eat from the bowl (as was the case with some small dogs), the tester fed the treat to the dog once the dog had clearly approached the correct bowl, and this was done on all consecutive training and test trials. The owner then took the dog back to the starting position, and the same procedure was repeated with the bowl and treat placed on the right side.

After this, the tester placed two identical bowls on the floor in front of her, approximately 95 cm apart from each other. Similar to the previous trials, the tester placed a piece of food inside the left-hand bowl while the dog was watching. The dog was released and allowed to eat the food (and to investigate the empty bowl). Both the owner and tester remained stationary and looked directly forwards, without giving the dog an indication about the correct direction. After this the owner collected the dog, returned to the starting position, and the same procedure was repeated with the treat placed in the bowl on the right-hand side. The test phase was started only if the dog successfully went to the correct bowl on four consecutive trials. (See Supplementary Video 3 for the training phase procedure.)

***Test Phase***

Dogs took part in six trials for each gesture (30 trials in total). (See Supplementary Video 4 for the test procedure for each of the five gestures.) Each dog received the gestures in the same order, starting from dynamic distal pointing. The percentage of correct responses (out of 30 trials) was calculated from all the gesture tests combined.

Bowls were baited in the same order for each dog. On the first trial, the food reward was placed in the left-hand bowl. Every other trial was baited to the right and every other to the left. (Trial 1: left, trial 2: right, trial 3: left, trial 4: right, etc.) In order to ensure dogs were not learning this pattern, the percentage of correct responses from the final 6 trials (gaze) were compared to the percentage of correct responses from the first 6 trials (dynamic distal pointing) using a two-tailed paired t-test. In conclusion, each dog received 15 trials where the bowl on the left side included the food reward, and 15 trials where the bowl on the right side included the food reward. This ensured that one side was not rewarded consecutively more than once and that all dogs received exactly the same order of rewarded locations.

Each of the 30 trials during the test phase was performed in the following way, with the only difference being the specific gesture used. The trial always started with the dog and owner facing the tester, who was standing 2―2.5 metres away. Similar to the training phase, if the dog was not looking in the direction of the tester, the tester said the dog’s name to get the dog’s attention while holding the two bowls in her hands. She showed a piece of food to the dog and placed it inside one of the bowls, which she held one in front of the other in her arms, so that there was no right or left side from the point of view of the dog Once the tester had placed the treat in one of the bowls, she then exchanged the bowls from hand to hand one or two times, so that the dog could not see which bowl contained the treat.

The bowls were placed on the floor simultaneously, 95cm apart from each other, identically to the training phase. Both bowls were an equal distance away from the dog’s starting position. The tester established eye contact with the dog and provided the gesture, making sure that the dog was looking. If the dog was not looking in the direction of the tester, the tester again said the dog’s name. At this point, there was no indication to the dog about which side was correct, and once the tester had the dog’s attention, she performed the gesture without talking. The gesture was not provided until the dog was looking at the tester. After the tester had provided the gesture, the owner released the dog. While the dog made their choice, both the owner and the experimenter were silent and stood still, looking directly forwards and avoiding any indication towards the correct bowl (apart from the given gesture). Throughout the trial, the tester remained standing in the middle-point between the two bowls, without making any movement towards either bowl (with the exception of ‘gaze’, where she took a small step away from the correct bowl).

The trial was marked as correct if the dog chose the baited bowl by putting their nose inside. The trial was marked as incorrect if the dog chose the un-baited bowl first, approached the tester only, or did not make a choice. In these cases, the dog was still allowed to eat the treat from the baited bowl if they decided to do so. If the dog went to the same bowl in two consecutive trials, the dog was then not allowed to eat the food from the baited bowl if an incorrect choice was made in the next trial. This was done to prevent the dog from forming a pattern or side bias. If the dog was not allowed to eat the treat after an incorrect choice, immediately afterwards the dog was provided with a simple warm-up trial, where the treat was placed in the previously incorrect bowl and the dog was allowed to eat it. This was done to ensure that the dog would feel that both bowls are ok to approach. If a dog became uncertain after reward omission (i.e., hesitant or delayed approach of either bowl), the reward was not removed any more for this dog, in order to maintain motivation for the task.

The gestures provided to the dog are described below in detail in the order in which they were performed. The procedure for each gesture was otherwise the same as described above.

***Dynamic Distal Pointing***

The procedure was performed as described above; the tester placed a treat into one of the bowls she was holding while the dog was watching. If necessary, she said the dog’s name to make sure the dog was looking. She then placed the bowls on the floor in front of her, one on the left and one on the right. While the dog was watching from the starting position, the tester pointed with an extended arm and index finger in the direction of the correct bowl for about 2 seconds. The tester was standing, and therefore the tip of her finger was approximately 40―70 cm away from the bowl, depending on the height of the tester. She used the arm closest to the correct bowl, while keeping the other arm flush with the side of her body. The tester kept her arm in the same position while the owner released the dog and while the dog was making their choice (Supplementary Figure 2). The arm was lowered only when the dog chose a bowl or when the trial ended. After this, the owner collected the dog back to the start position, and another five trials were performed.


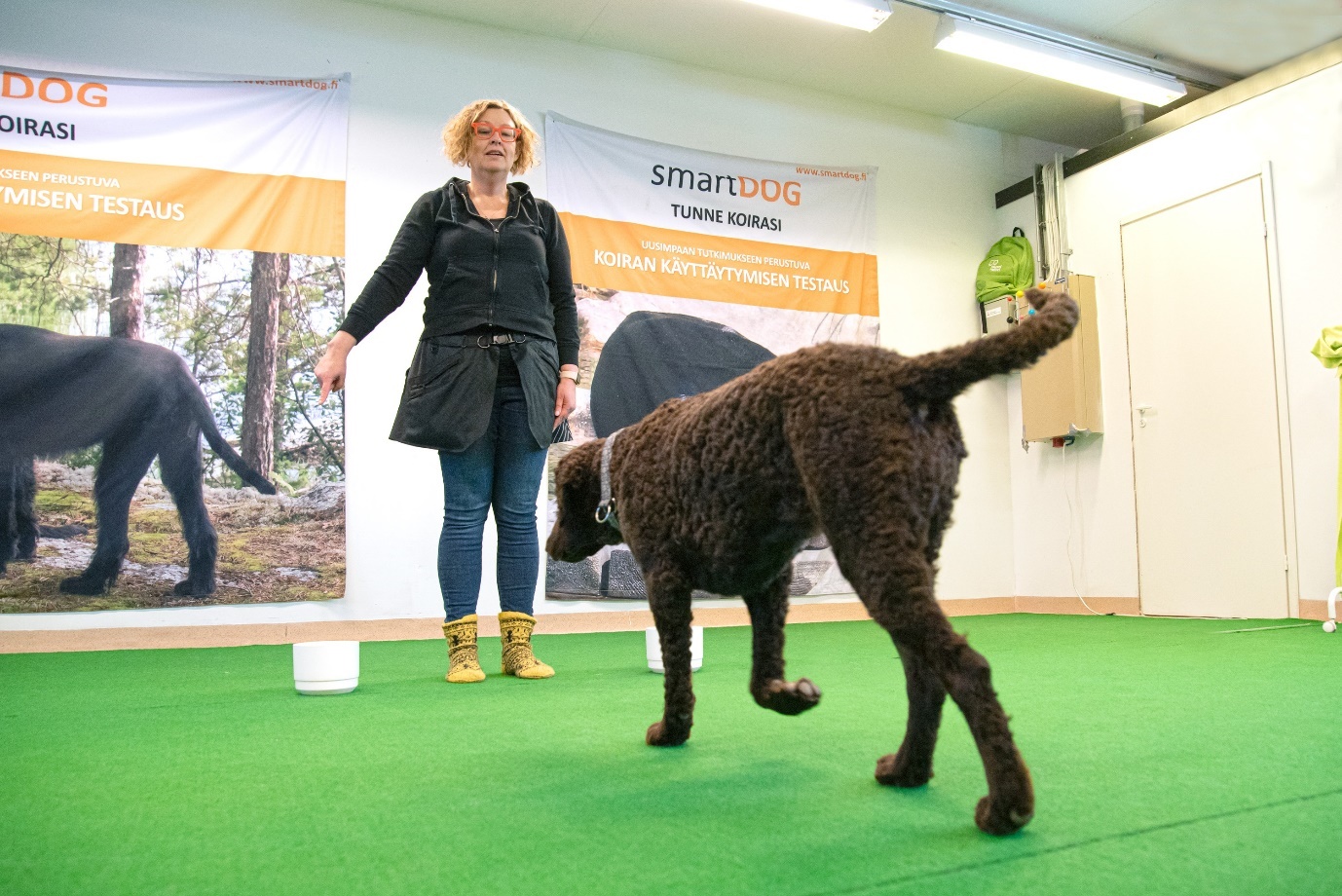


**Supplementary Figure 2**. Test phase for Dynamic Distal Pointing. The dog has been released to make a choice between two bowls, one of which the tester is pointing towards. Image by Mainossatama Oy.

***Momentary Distal Pointing***

The procedure was otherwise similar to the dynamic distal pointing, but after pointing at the correct bowl for a duration of 2 seconds, the tester lowered her arm to the starting position flush with her body and waited another 2 seconds before the dog was released. She remained in this neutral position until the end of the trial, while the dog chose a bowl. The owner then collected the dog and returned to the starting position, and the procedure was repeated another five times until moving on to the next gesture.

***Dynamic Proximal Foot Pointing***

The tester held her arms behind her back and pointed with an extended foot in the direction of the correct bowl for about 2 seconds before the dog was released. The tester used the leg closest to the correct bowl and placed the tip of her foot on the ground directly behind the correct bowl. She kept her face and body facing directly forward. The dog was released while the tester continued pointing with her foot, and the foot was returned to its starting position when the trial ended. The dog chose a bowl while the tester was still pointing with her foot, after which the owner collected the dog to the starting position. Five more trials were performed with this gesture.

***Dynamic Cross-forward Pointing***

The tester pointed with her contralateralhand (opposite to the correct bowl) towards the correct bowl while rotating her shoulders in the same direction. The tester sustained this position while the dog was released and made their choice, and she returned her arm to the starting position when the trial ended. The owner collected the dog after the trial, and five more trials were performed.

***Gaze***

The tester took a small step away from the correct bowl, so that she stood about 60 cm away from it, closer to the incorrect bowl. While facing the dog, she turned her head and eyes towards the correct bowl, gazing at it, keeping the rest of her body facing forwards and her arms flush with the side of her body. She then alternated her gaze between the dog and the bowl three times, so that she looked at each for about 1 second at a time. The dog was released while the tester was gazing at the correct bowl. She continued her gaze towards the bowl until the trial had ended and the dog had made their choice. The dog was then returned to the starting position and the trial was repeated another five times.

**V-detour**

After the gesture tests, the owner took the dog outside for a small (approximately 2―5-minute) break, while the tester set up the V-fence. The V-detour has been used in canine cognitive research to investigate spatial problem-solving ability^14^. The dog has to detour around a transparent V-shaped fence to access a food reward which is placed on the other side (Supplementary Fig. 3, Supplementary Video 5). Since the dog is required to move away from the visible treat to access it, the task is often considered to also measure inhibitory control.


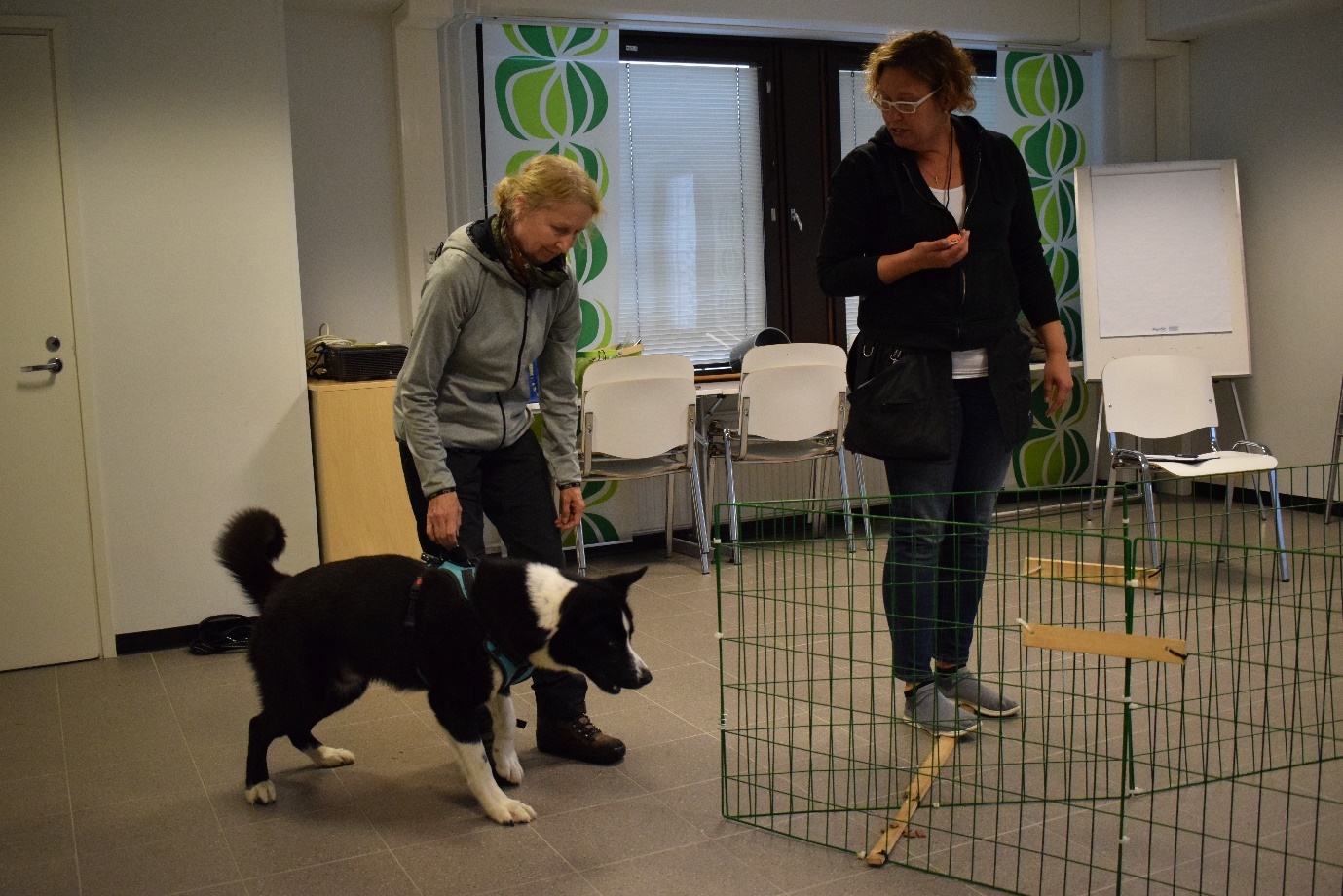


**Supplementary Figure 3.** The V-detour task. Food has been placed on the wooden board inside the V-shaped fence, and the dog has been released to attempt to access the food. Image by Minna Sirviö.

The V-shaped fence consisted of two sides which were made out of compost fence panels, which were attached at an approximately 70° angle. Both sides were 180 cm in length and 69 cm in height. The fence sides were attached to a wooden board at the bottom, and the tester stood on top of the board outside the fence to prevent the dog from moving the fence around. The fence was placed so that the intersecting corner of the V was facing the door where the dog came in from, with the ‘sharp’ edge pointing toward the dog and owner. The owner and dog waited about 40 cm away from the intersecting angle of the V-shaped fence. If the dog was already not looking at the tester, she said the dog’s name to attract their attention. The tester showed the dog several treats (or a toy) and placed them inside the fence, close to the intersecting angle on top of the wooden board, while standing outside of the fence herself. She made sure that the dog was aware of the treats by pointing at them and saying “look”. After this, the tester stood still, without making any more indication toward the food. The owner released the dog while the dog was looking at the food.

While the dog was attempting to solve the task, the tester stood still outside the fence. The owner and tester did not talk to the dog or move their body or arms in the correct direction. If the dog remained where it was or stared at the owner or tester for a long period, the owner encouraged the dog by using the word which indicated to the dog that it was allowed to take the food (e.g., “ok”). This word was repeated if necessary. The number of seconds taken to solve the task was measured with a stopwatch starting from the moment when the dog was released and ending when the dog touched the food. If the dog was not able to solve the task within 3 minutes, the trial was terminated, and the dog’s result was marked as unsuccessful.

Two variables were used for measuring success in the V-detour task. First, we compared dogs which solved the task within 180 s and dogs which failed to solve the task within this time. Second, we used standard multiple regression to analyse time (s) to solve the V-detour for the dogs which solved the task within the given time period.

**Unsolvable Task**

The unsolvable task has been used in canine cognitive research to assess persistence, problem-solving behaviour, human-directed communication, and social cognition^27^. In our version of this task, the dog was presented with four solvable trials, after which the task became impossible to solve (Supplementary Fig. 4, Supplementary Video 6). The dog then had three opportunities for action: a) attempting to solve the problem independently, b) initiating social contact with a human, which has been interpreted as help-seeking behaviour, or c) abandoning the task and doing something else. The amount of time the dog spent on each behaviour during a 2-minute period was measured.


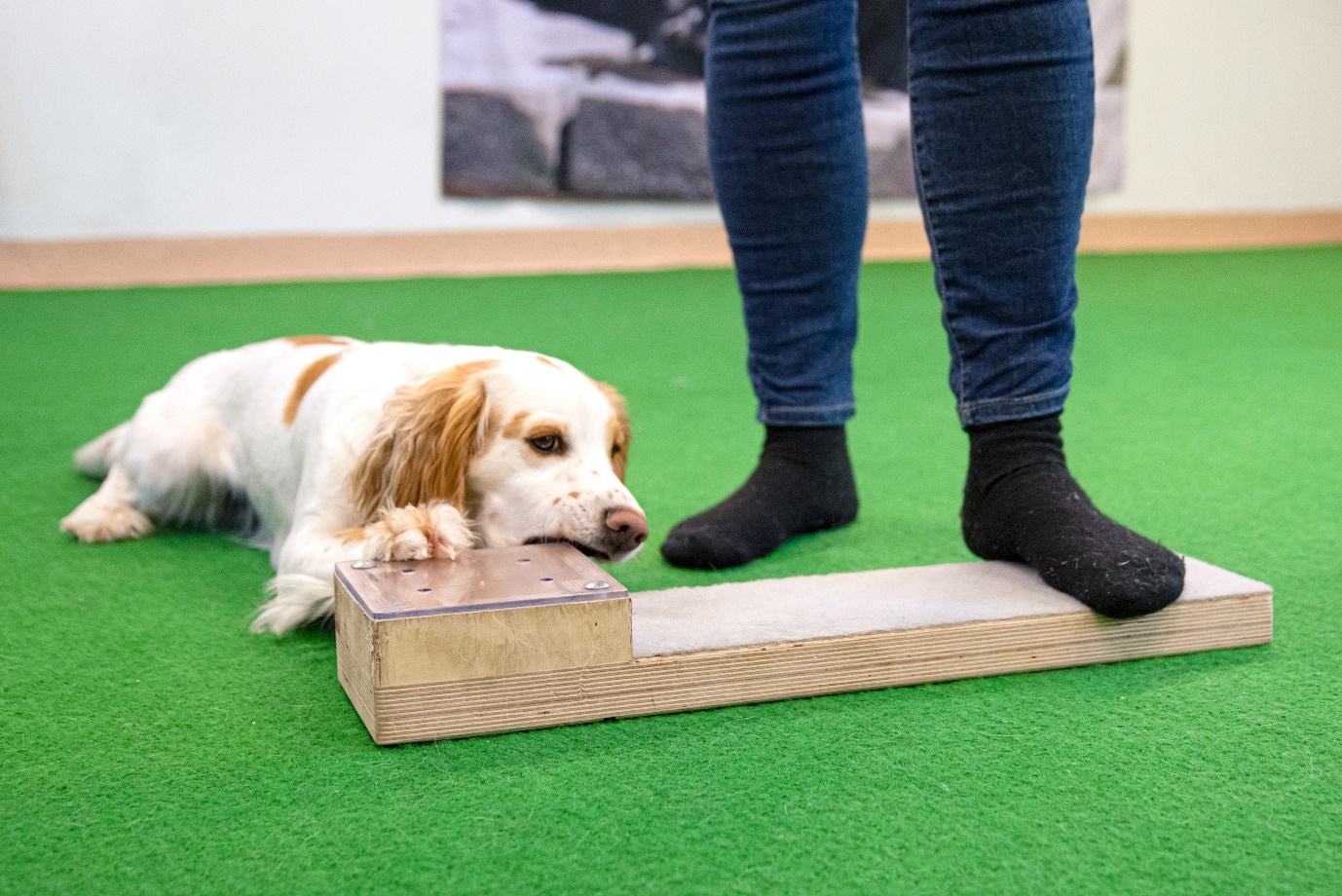


**Supplementary Figure 4**. Test phase for the Unsolvable Task. The dog is attempting to solve the problem independently. Image by Mainossatama Oy.

The dog was presented with a problem-solving box (Supplementary Fig. 4) placed on the floor. The dimensions of the box were 15 cm × 15 cm × 6 cm, 15 cm × 15 cm × 9 cm, or 20 cm × 20 cm × 9 cm for medium to large dogs and 11.5 cm × 11.5 cm × 7 cm for small dogs. For medium and large dogs, the box was wooden with a plastic, transparent lid. For small dogs, the box was transparent plastic. The top of the box had several small holes to allow the dog to smell the food inside. The box was attached to a wooden board on the floor, which the tester held in place with her foot during the trials.

During training trials, the dog was taught to access the food inside the box by moving the plastic lid with their muzzle or paw. The owner held the dog 1―1.5 metres away from the box, and the tester showed the dog a treat and placed it inside the box. If the dog was not looking in the direction of the tester, she said the dog’s name to attract their attention. The tester covered the treat very slightly with the lid, so that the dog only needed to push the lid lightly to access the treat. After this, the dog was released and was allowed to access the food reward. The owner then collected the dog and returned to the starting position. This was repeated another three times, with the difficulty of the trials increasing gradually, until the lid was placed so that it covered the food completely. If necessary, the difficulty of the task was decreased until the dog was successful, and additional trials were performed until the dog was successful. Both the owner and the tester encouraged the dog verbally to access the treats during the training trials, but they offered no other help.

Once the dog successfully accessed the treat when the lid completely covered the treat, the test trial was begun. The owner held the dog 1―1.5 metres away from the box, and the tester showed the dog several treats and placed them inside the box (and, if necessary, saying the dog’s name to attract their attention). She then turned away from the dog and secured the lid in place so that it could not be opened. She then placed the box on the floor in front of the dog, and the owner released the dog. Both owner and experimenter remained standing, quiet and still, looking only at the box during the subsequent 2-minute period. They did not talk or look at the dog or make any movement toward the dog or the box.

The tester used two silent stopwatches to monitor the time the dog spent on each behaviour. These were coded as follows:

*Human-directed behaviour:* The dog looks at either the owner or the tester, alternates between looking at a person and the box, or performs previously learned tasks (e.g., sitting, lying down) while looking at the person.

*Independent problem-solving:* The dog sniffs, looks at, or touches the apparatus with nose, muzzle, or paw, or manipulates the box in any way.

*Abandoning task:* The dog does not engage in human-directed or problem-solving behaviour, doing something else instead (such as sniffing the ground, exploring the environment, self-grooming, or looking away from the box and humans).

Three variables were used to measure the dogs’ behaviour during the unsolvable task: complete independence, human-directed behaviour, and abandoning the task. Since human-directed behaviour and independent behaviour were strongly negatively correlated (when dogs were performing one behaviour, they could not perform the other, and only a small number of dogs abandoned the task), only human-directed behaviour was analysed. Some dogs were completely independent in this task (spending 0 % of their time on human-directed behaviour), while most dogs showed at least some human-directed behaviour. Thus, the data were bimodally distributed, and therefore dogs were divided into two groups: those which spent 0 % of their time on human-directed behaviour (completely independent group), and those which spent over 0 % of their time on human-directed behaviour (human-directed group). Differences between these two groups were then analysed. For the human-directed group, standard multiple regression was used to analyse the percentage of time dogs spent on human-directed behaviour. To determine which dogs abandoned the task more readily than others, we analysed the time dogs spent on ‘abandoning the task’, i.e., not attempting to solve the task and not engaging in human-directed behaviour. As a large majority of dogs spent 0 % of their time abandoning the task, the dogs were divided into two groups: those which spent 0% of their time abandoning the task (‘persistent’ group), and those which spent over 0 % of their time abandoning the task (‘abandoned task’ group).

**Logical reasoning**

This test aimed to measure the dog’s ability to make inferences based on exclusion. The dog could see that one of two bowls was empty, and they had to infer that the treat was hidden under the other bowl. Similar tests for measuring logical reasoning have been used in previous studies with dogs^28-32^. The task was a modified version of the procedure by Erdőhegyi et al.^29^

The tester sat on a chair or on the floor, facing the dog. The dog waited with its owner about 1 m away, facing the tester. Two identical, opaque plastic bowls (height 14 cm, diameter 12 cm) were placed upside down in front of the tester at arm’s length, one on the left and one on the right side, approximately 50 cm apart from each other. A white sheet of plastic or paper was placed underneath both bowls, in order to make the food more visible. In each trial, a piece of food was placed under one of the bowls. The order of baiting was the same for each dog; during the first trial the left-hand bowl was baited, after which every other trial was baited to the right and every other trial to the left (trial 1: left, trial 2: right, trial 3: left, etc.). Therefore, dogs received an equal number of trials with the reward hidden under the left and the right bowl. (In the training phase, 2 trials were rewarded to the left and 2 to the right, and in the test phase, 3 trials were rewarded to the left and 3 to the right.)

***Training Phase***

The aim of the training phase was to familiarize the dog with the testing procedure, to allow the dog to experience that the treat was always under only one of the bowls and, most importantly, to encourage the dog to use visual cues. (See Supplementary Video 7 for the procedure of the training phase.) The tester showed a piece of food to the dog and placed it under the left bowl. If the dog was not looking in the direction of the tester, she said the dog’s name to attract their attention before hiding the treat. When the tester had hidden the treat, she lifted both bowls up, about 30 cm above the floor, and kept them there for about one second, making sure the dog was watching. The dog could therefore see where the treat was hidden. The tester kept her gaze forward, not looking at either bowl. Again, if the dog was not looking, the tester said the dog’s name before lifting the bowls up. When the dog was still watching, the tester placed the bowls back on the floor, one covering the treat, and placed her arms on her lap. The dog was released, and the tester and owner remained stationary, body and gaze directed forward, without providing the dog with any help as to where the food was hidden. If the dog approached the correct bowl, the tester lifted the bowl and allowed the dog to eat the treat. During the training phase, if the dog approached the incorrect bowl, the bowl was lifted to show the dog it was empty, the food was removed from under the correct bowl, and the trial was repeated. If the dog chose the correct bowl, the owner collected the dog, and the trial was repeated with the food reward hidden under the bowl on the right.

After this, two trials identical to the previous ones were performed, but the tester used a visual barrier to prevent the dog from seeing the baiting. The tester placed an A4-sized writing pad in front of the left bowl, so that the view of the bowl was occluded from the dog. While holding the writing pad in front of the bowl, the tester placed a treat under the left bowl. She then placed the writing pad in front of the right bowl, and sham-baited the bowl. The writing pad was then removed, the tester showed the dog her empty hands, and the trial was continued as before; the tester lifted both bowls up to show the dog where the treat was, after which they were placed back down, and the dog was released. When the dog had eaten the treat, the owner collected the dog back to the starting position. The same was repeated on the right-hand side; the tester placed the writing pad in front of the left-hand bowl and sham-baited the bowl. She then placed the writing pad in front of the right-hand bowl and baited it with a treat. When the dog correctly performed all four training trials, the test phase was initiated. If the dog made a mistake during the training phase (which very rarely happened), the same trial was repeated.

***Test Phase***

The test phase consisted of six trials. These were performed identically to the last two pre-training trials, except the tester now lifted only the empty bowl, while holding her other hand on top of the baited bowl. This was done to prevent the dog from simply choosing the bowl which the tester was touching. If the dog approached the baited bowl, both bowls were lifted, and the dog was allowed to eat the treat. If the dog did not approach either bowl but continued looking at one of the bowls, the tester verbally encouraged the dog (by saying “ok” or similar). If the dog chose the incorrect bowl, the tester lifted the bowl to show it was empty, and then lifted the baited bowl and allowed the dog to eat the treat. If the dog approached the tester or did not choose either bowl, this was marked as an incorrect trial. (See Supplementary Video 8 for the procedure of the test phase.)

Two variables were used to measure logical reasoning of dogs: percentage of correct responses and understanding of the task. The percentage of correct responses was divided into three groups, since dogs making incorrect choices may have used different strategies, resulting in different scores which do not necessarily reflect the abilities of the dogs. The groups were divided as follows:

1) 0-50 % correct

2) 51-82 % correct

3) 83-100 % correct

In addition, the tester also evaluated whether the dog had understood the task and was successfully making inferences based on exclusion. The dog was considered to have understood the task if: a) at least 4 out of 6 trials were correct, or b) the final 2―3 trials were correct (suggesting that the dog had learned the task during the previous trials). If it was unclear whether the dog had understood the task, an additional trial was performed on the side less often chosen by the dog.

**Memory vs Gesture**

Previous studies have shown that dogs are more likely to choose an empty bowl out of two choices if a human points towards it, even when they have seen that the other bowl has food in it^20,33-35^. The procedure for this test was otherwise similar to the gesture test, but instead of gesturing towards the baited bowl, the tester gestured towards the empty bowl. Therefore, the dog could either choose the bowl where they had seen the tester place the treat, or the bowl which the tester was gesturing towards. The tester used the gesture which the dog had been most successful with – this was usually the dynamic distal point. The bowls remained on the floor throughout the test, and the treat was placed in one of the bowls while the dog was watching. The test consisted of two trials, with the first trial always baited to the left and the second trial baited to the right. The dog’s reliance on the human’s gestures was measured based on the number of times the dog approached the empty, gestured bowl instead of the baited bowl. The trial was marked as ‘correct’ if the dog approached (put their nose inside) the baited bowl, and ‘incorrect’ if they chose the empty bowl, approached the tester, or failed to make a choice. (See Supplementary Video 9 for the procedure.)

A very small number of dogs chose the empty, gestured container on both trials, and therefore these dogs were combined into a group with dogs which chose the empty container on one trial. This resulted in two groups of dogs: those which chose the empty container on 1―2 trials (relied on gesture), and those which chose the baited container on both trials (relied on memory).

**Memory**

The aim of this test was to measure the duration of the dogs’ short-term memory, and it was based on the procedure by Fiset et al.^36^ In this test, the dog was required to remember the location of a food treat, which was hidden under one of three bowls, with an increasing duration of waiting time.

Three identical, opaque plastic bowls (height 14 cm, diameter 12 cm) were placed upside down on the floor in a straight line, about 1 m apart from each other. A piece of food (or a toy) was placed under one of the bowls in each trial. The owner sat on a chair 3 m away from the middle bowl with the dog in front of her, both facing the bowls. The owner was advised to release the dog only when the dog was looking towards the bowls. If the dog was looking elsewhere, the owner waited until the dog was facing the bowls before releasing the dog.

***Training Phase***

The training phase was performed in order to familiarize the dog with the testing procedure. The tester showed the dog a piece of food (or a toy) and, if necessary, attained the dog’s attention by saying the dog’s name and “look”. While the dog was watching, the tester walked over to the bowl on the right side (from the dog’s point of view) and placed the food under the bowl, while saying the dog’s name and “look” to maintain attention on her. She then touched and clearly pointed at the bowl to indicate to the dog that the treat was hidden underneath and said “look” again. The tester remained standing behind the baited bowl, hands by her sides, and the dog was released when the dog was looking at the bowls. While the dog made their choice, the tester remained stationary, not talking to the dog or making any movement toward the correct bowl. If the dog approached the correct bowl, the tester lifted it and allowed the dog to eat the food, after which the owner returned the dog to the starting position. If the dog approached one of the empty bowls during the training phase, the owner was advised to collect the dog calmly and quietly and return to the starting point. The same procedure was repeated for the middle bowl and the left bowl. If the dog chose the correct bowl in all three trials, they continued to the next phase. If necessary, trials were repeated if the dog chose the incorrect bowl.

The next three trials were similar to the previous ones, but after the tester had baited one of the bowls, she walked to a spot behind the owner and dog and stood there with her back facing the bowls. The owner released the dog when the dog was looking in the direction of the bowls (Supplementary Video 10). If the dog made the correct choice, the bowl was lifted and the dog was allowed to eat the treat, after which the dog was returned to the starting position. If the dog made an incorrect choice, the owner collected the dog, and the trial was repeated once.

When the dog was successful on each of these three trials, they were presented with a trial including a visual barrier. A treat was placed under the right-hand bowl, after which the tester moved avisual barrier (a screen on wheels) calmly between the dog and the bowls, preventing visual access to the bowls. The barrier was kept in place for a duration of 3 seconds, after which it was moved back to the side, the tester moved to a spot behind the owner, and the dog was released when looking towards the bowls. This was done to familiarize the dog with the barrier and make sure the dog was not fearful of it. If the dog was clearly afraid of the barrier, the owner was advised to instead turn her back to the bowls during the waiting period, and make sure the dog was turned away as well. In these cases, the trial was repeated once more without the barrier, before continuing onto the test phase.

***Test Phase***

Once the dog had passed the training trials, the test trials began. These were similar to the final training trial; the tester baited one of the bowls while the dog was watching (while saying the dog’s name and “look”), moved the visual barrier in front of the dog and then went to sit at the back of the room, behind the dog and owner. The difference was that the time between baiting the bowl and releasing the dog was increased in each trial. The test phase included four trials, starting from a waiting period of 1 minute. This was increased first to 1.5 minutes during the second trial, then to 2 minutes during the third trial, and finally to 2.5 minutes during the fourth trial. During the waiting period the tester remained sitting on a chair quietly behind the owner and dog. While waiting, the owner was allowed to talk to and try to calm the dog if necessary. The order of baiting the bowls was the same for each dog: 1) middle, 2) left, 3) right, and 4) middle. When the waiting period was over, the tester removed the visual barrier and then sat behind the owner and dog to the side. The owner released the dog when the dog was looking in the direction of the bowls. If the dog chose the correct bowl, the tester lifted the bowl and allowed the dog to eat the food reward. If the dog chose the incorrect bowl during the test phase, the tester lifted the bowl to show that it was empty and the dog was then allowed to eat the treat from under the baited bowl.

The choice was marked as correct if the dog approached the correct bowl directly, or if the dog approached the correct bowl first but then changed direction at the last moment. The trial was marked as incorrect if the dog approached one of the empty bowls directly, or if the dog started to approach an empty bowl and then changed direction at the last moment and went to the correct bowl. The trial was also marked as incorrect if the dog made no choice or approached the tester instead. If the dog approached the same bowl in each trial, the dog was given a score of 1 (out of 4). (See Supplementary Video 11 for the procedure of the test phase.)

Number of correct trials (out of a total of 4) was calculated for each dog. Since a very small number of dogs had a score of 0, these dogs were combined into a group with dogs that had a score of 1.

**Results**

| Greeting new person |  | Fearful |  |  | Indifferent |  |  | Overexcited |  |
| --- | --- | --- | --- | --- | --- | --- | --- | --- | --- |
| Variable | OR | 95 % CI | P-value | OR | 95 % CI | P-value | OR | 95 % CI | P-value |
| Breed |  |  |  |  |  |  |  |  |  |
| Golden Retriever | 0.3 | 0.08 to 1.11 | 0.86 | 0.48 | 0.22 to 1.04 | 0.76 | 0.34 | 0.15 to 0.77 | 0.12 |
| Hovawart | 0.47 | 0.1 to 2.23 | 1 | 2.53 | 1.22 to 5.24 | 0.16 | 0.25 | 0.07 to 0.92 | 0.43 |
| English Cocker Spaniel | 0.65 | 0.2 to 2.14 | 1 | 0.83 | 0.37 to 1.82 | 1 | 0.6 | 0.26 to 1.37 | 1 |
| German Shepherd | 0.7 | 0.23 to 2.14 | 1 | 1.90 | 0.98 to 3.69 | 0.68 | 0.21 | 0.07 to 0.65 | 0.07 |
| Australian Shepherd | 1.08 | 0.35 to 3.34 | 1 | 1.07 | 0.47 to 2.42 | 1 | 0.25 | 0.07 to 0.88 | 0.37 |
| Finnish Lapphund | 1.27 | 0.46 to 3.5 | 1 | 1.44 | 0.7 to 2.98 | 1 | 0.28 | 0.09 to 0.85 | 0.3 |
| Border Collie | 1.43 | 0.62 to 3.28 | 1 | 1.42 | 0.76 to 2.65 | 1 | 0.3 | 0.13 to 0.7 | 0.07 |
| Australian Kelpie | 2.04 | 0.56 to 7.49 | 1 | 1.4 | 0.49 to 4.04 | 1 | 2.99 | 1.2 to 7.44 | 0.22 |
| Mixed Breed | 2.33 | 1.11 to 4.88 | 0.3 | 1.86 | 1.06 to 3.28 | 0.37 | 0.35 | 0.16 to 0.76 | 0.1 |
| Belgian Shepherd Malinois | 3.72 | 1.02 to 13.51 | 0.55 | 4 | 1.47 to 10.9 | 0.08 | 1.15 | 0.31 to 4.25 | 1 |
| Spanish Water Dog | 4.54 | 1.95 to 10.55 | **0.005** | 3.05 | 1.51 to 6.14 | **0.02** | 0.09 | 0.01 to 0.69 | 0.25 |
| Shetland Sheepdog | 5.31 | 2.13 to 13.22 | **0.004** | 1.25 | 0.51 to 3.08 | 1 | 0.14 | 0.02 to 1.09 | 0.72 |
| Sex | 0.9 | 0.59 to 1.38 | 0.63 | 1.18 | 0.86 to 1.62 | 0.3 | 1.27 | 0.81 to 1.98 | 0.29 |
| Age | 0.99 | 0.98 to 1 | **0.05** | 1.01 | 1 to 1.01 | 0.18 | 0.98 | 0.97 to 0.99 | **0.002** |

**Supplementary Table 3.** Parameter estimates for the multinomial regression for greeting an unknown person (n = 934), with the Labrador Retriever as the reference breed. Each greeting score is compared to the ‘friendly’ greeting (score 6). Breeds have been ordered based on odds ratios for ‘fearful’ response, with lowest fearfulness at the top and highest fearfulness at the bottom. P-values of breeds have been Bonferroni-corrected, and significant p-values are in bold.

| Variable | B | 95 % CI for B | Beta | P-value |
| --- | --- | --- | --- | --- |
| Breed |  |  |  |  |
| Belgian Shepherd Malinois | 6.83 | 3.3 to 10.37 | 0.14 | **0.002** |
| English Cocker Spaniel | 3.61 | 0.98 to 6.23 | 0.104 | 0.09 |
| German Shepherd | 2.66 | 0.27 to 5.05 | 0.09 | 0.38 |
| Australian Kelpie | -1.15 | -4.4 to 2.1 | -0.03 | 1 |
| Australian Shepherd | -1.59 | -4.38 to 1.2 | -0.04 | 1 |
| Border Collie | -1.66 | -3.75 to 0.43 | -0.07 | 1 |
| Spanish Water Dog | -2.25 | -4.8 to 0.31 | -0.07 | 1 |
| Mixed Breed | -3.63 | -5.95 to -1.3 | -0.12 | **0.03** |
| Golden Retriever | -3.66 | -6.18 to -1.15 | -0.11 | **0.05** |
| Finnish Lapphund | -4.92 | -7.5 to -2.33 | -0.15 | **0.002** |
| Shetland Sheepdog | -5.02 | -7.82 to -2.23 | -0.14 | **0.005** |
| Hovawart | -6.01 | -8.65 to -3.37 | -0,17 | **<0.001** |
| Sex | -0.48 | -1.65 to 0.68 | -0.03 | 0.42 |
| Age | -0.02 | -0.04 to 0.01 | -0.04 | 0.23 |

**Supplementary Table 4.** Parameter estimates for the multiple regression for activity level (n = 759) during the COGNITION test battery (measured in FitBark activity points), with the Labrador Retriever as the reference breed. Breeds have been ordered based on B-values, with highest activity levels at the top and lowest activity levels at the bottom. Bonferroni-corrected p-values for breeds are reported. Significant p-values are in bold.

| Variable | OR | 95 % CI | P-value |
| --- | --- | --- | --- |
| Breed  English Cocker Spaniel | 2.17 | 1.14 to 4.13 | 0.23 |
| Belgian Shepherd Malinois | 1.73 | 0.74 to 4.09 | 1 |
| Australian Kelpie | 1.29 | 0.63 to 2.65 | 1 |
| German Shepherd | 1.24 | 0.69 to 2.21 | 1 |
| Golden Retriever | 0.86 | 0.5 to 1.5 | 1 |
| Australian Shepherd | 0.65 | 0.34 to 1.24 | 1 |
| Spanish Water Dog | 0.61 | 0.35 to 1.04 | 0.84 |
| Mixed Breed | 0.58 | 0.37 to 0.91 | 0.2 |
| Border Collie | 0.57 | 0.34 to 0.95 | 0.36 |
| Hovawart | 0.48 | 0.23 to 1 | 0.61 |
| Finnish Lapphund | 0.39 | 0.22 to 0.71 | **0.02** |
| Shetland Sheepdog | 0.22 | 0.12 to 0.42 | **<0.001** |
| Sex | 0.97 | 0.75 to 1.26 | 0.82 |
| Age | 1 | 0.99 to 1 | 0.17 |

**Supplementary Table 5.** Parameter estimates for the multiple regression for exploration of a novel environment (n = 820), with the Labrador Retriever as the reference breed. Breeds are ordered based on odds ratios, with highest curiosity/boldness at the top and lowest curiosity/boldness at the bottom. P-values for breeds are Bonferroni-corrected, and significant p-values are in bold.

| Variable | B | 95 % CI for B | Beta | P-value |
| --- | --- | --- | --- | --- |
| Breed |  |  |  |  |
| Border Collie | -1.9 | -2.49 to -1.31 | -0.24 | **<0.001** |
| Mixed Breed | -1.79 | -2.32 to -1.25 | -0.26 | **<0.001** |
| Australian Shepherd | -1.33 | -2.09 to -0.56 | -0.12 | **0.008** |
| Golden Retriever | -1.12 | -1.79 to -0.46 | -0.12 | **0.01** |
| Shetland Sheepdog | -1.11 | -1.88 to -0.33 | -0.1 | 0.06 |
| Spanish Water Dog | -1.01 | -1.67 to -0.34 | -0.11 | **0.04** |
| English Cocker Spaniel | -0.75 | -1.45 to -0.04 | -0.07 | 0.47 |
| Australian Kelpie | -0.71 | -1.57 to 0.15 | -0.05 | 1 |
| Finnish Lapphund | -0.67 | -1.38 to 0.04 | -0.06 | 0.792 |
| Hovawart | -0.59 | -1.37 to 0.19 | -0.05 | 1 |
| German Shepherd | -0.48 | -1.12 to 0.15 | -0.05 | 1 |
| Belgian Shepherd Malinois | -0.37 | -1.17 to 0.423 | -0.03 | 1 |
| Sex | -0.45 | -0.75 to -0.14 | -0.09 | **0.004** |
| Age | 0.02 | -0.01 to 0.02 | 0.13 | **<0.001** |

**Supplementary Table 6.** Parameter estimates from the multiple regression for percentage of incorrect trials in the cylinder test (n = 992), with the Labrador Retriever as the reference breed. Reflected and square root transformed scores were used as the dependent variable. Therefore, the regression coefficients should be interpreted accordingly. Breeds are ordered based on B-values, with highest success (high inhibitory control) at the top and lowest success (low inhibitory control) at the bottom. P-values for breeds are reported with Bonferroni correction. Significant p-values are in bold.

| Variable | B | 95 % CI for B | Beta | P-value |
| --- | --- | --- | --- | --- |
| Breed |  |  |  |  |
| Belgian Shepherd Malinois | 0.95 | -4.36 to 6.27 | 0.01 | 1 |
| Australian Kelpie | 0.83 | -4.1 to 5.76 | 0.01 | 1 |
| Border Collie | -0.12 | -3.35 to 3.1 | -0.003 | 1 |
| Golden Retriever | -2.03 | -5.74 to 1.67 | -0.04 | 1 |
| Hovawart | -2.21 | -6.31 to 1.9 | -0.04 | 1 |
| Spanish Water Dog | -4.49 | -8.37 to -0.61 | -0.09 | 0.29 |
| Shetland Sheepdog | -4.97 | -9.3 to -0.65 | -0.09 | 0.29 |
| English Cocker Spaniel | -5.27 | -9.17 to -1.36 | -0.1 | 0.1 |
| Australian Shepherd | -5.46 | -9.74 to -1.17 | -0.1 | 0.16 |
| Mixed Breed | -6.6 | -10.11 to -3.09 | -0.15 | **0.003** |
| German Shepherd | -6.69 | -10.39 to -2.99 | -0.14 | **0.005** |
| Finnish Lapphund | -8.02 | -11.98 to -4.06 | -0.15 | **<0.001** |
| Sex | -1.43 | -3.21 to 0.35 | -0.06 | 0.12 |
| Age | 0.02 | -0.03 to 0.06 | 0.03 | 0.45 |

**Supplementary Table 7.** Parameter estimates from the multiple regression for percentage of correct trials in the gesture tests (n = 831), with the Labrador Retriever as the reference breed. P-values for breeds have been Bonferroni-corrected, and breeds have been ordered based on B-values, with highest success at the top and lowest success at the bottom. Significant p-values are in bold.

| Variable | B | 95 % CI for B | Beta | P-value |
| --- | --- | --- | --- | --- |
| Breed |  |  |  |  |
| Belgian Shepherd Malinois | -0.22 | -0.38 to -0.05 | -0.1 | 0.11 |
| Border Collie | -0.21 | -0.33 to -0.09 | -0.14 | **0.01** |
| Australian Shepherd | -0.21 | -0.37 to -0.05 | -0.1 | 0.12 |
| Australian Kelpie | -0.18 | -0.36 to 0.002 | -0.07 | 0.62 |
| German Shepherd | -0.17 | -0.3 to -0.04 | -0.1 | 0.13 |
| Mixed Breed | -0.11 | -0.23 to 0.001 | -0.08 | 0.61 |
| Finnish Lapphund | -0.07 | -0.22 to 0.08 | -0.04 | 1 |
| Spanish Water Dog | -0.06 | -0.2 to 0.08 | -0.03 | 1 |
| Shetland Sheepdog | -0.04 | -0.2 to 0.12 | -0.02 | 1 |
| Hovawart | -0.03 | -0.19 to 0.14 | -0.01 | 1 |
| English Cocker Spaniel | -0.03 | -0.18 to 0.12 | -0.01 | 1 |
| Golden Retriever | 0.09 | -0.06 to 0.23 | 0.04 | 1 |
| Sex | -0.05 | -0.11 to 0.01 | -0.05 | 0.12 |
| Age | 0.001 | 0 to 0.003 | 0.05 | 0.139 |

**Supplementary Table 8.** Parameter estimates from the multiple regression for time (s) taken to solve the V-detour (out of 180 s), for dogs which successfully solved the task within the given time period (n = 863). The Labrador Retriever was used as the reference breed. Log transformed scores were used as the dependent variable. Breeds have been ordered based on B-values, with fastest breeds at the top and slowest breeds at the bottom. P-values of breeds have been Bonferroni-corrected. Significant p-values are in bold.

| Variable | OR | 95 % CI | P-value |
| --- | --- | --- | --- |
| Breed |  |  |  |
| Golden Retriever | 2.86 | 0.34 to 24.21 | 1 |
| Spanish Water Dog | 2.58 | 0.3 to 21.89 | 1 |
| English Cocker Spaniel | 2.41 | 0.28 to 20.54 | 1 |
| Shetland Sheepdog | 1.85 | 0.22 to 15.8 | 1 |
| Australian Kelpie | 1.45 | 0.17 to 12.51 | 1 |
| Australian Shepherd | 0.96 | 0.19 to 4.94 | 1 |
| Border Collie | 0.64 | 0.2 to 2.04 | 1 |
| Finnish Lapphund | 0.56 | 0.15 to 2.06 | 1 |
| Hovawart | 0.38 | 0.1 to 1.43 | 1 |
| Mixed Breed | 0.36 | 0.13 to 0.96 | 0.5 |
| Belgian Shepherd Malinois | 0.26 | 0.08 to 0.87 | 0.35 |
| German Shepherd | 0.24 | 0.08 to 0.69 | 0.1 |
| Sex | 1.92 | 1.1 to 3.35 | **0.02** |
| Age | 1 | 0.98 to 1.01 | 0.44 |

**Supplementary Table 9.** Parameter estimates from the logistic regression for comparing the human-directed (over 0 % out of a total of 120 s spent on human-directed behaviour) and completely independent (0% of time spent on human-directed behaviour) groups in the unsolvable task (n = 969), with the Labrador Retriever as the reference breed. Breeds are ordered based on odds ratios, with the most human-directed breeds at the top and the most independent breeds at the bottom. P-values for breeds have been Bonferroni-corrected. Significant p-values are in bold.

| Variable | B | 95 % CI for B | Beta | P-value |
| --- | --- | --- | --- | --- |
| Breed |  |  |  |  |
| Australian Kelpie | 16.07 | 7.05 to 25.09 | 0.12 | **0.006** |
| Golden Retriever | 11.39 | 4.49 to 18.3 | 0.12 | **0.01** |
| Australian Shepherd | 10.93 | 2.91 to 18.96 | 0.1 | 0.1 |
| Border Collie | 8.29 | 2.05 to 14.54 | 0.1 | 0.11 |
| English Cocker Spaniel | 0.23 | -7.14 to 7.6 | 0.002 | 1 |
| Shetland Sheepdog | -0.44 | -8.55 to 7.67 | -0.004 | 1 |
| Mixed Breed | -1.85 | -7.59 to 3.9 | -0.03 | 1 |
| Belgian Shepherd Malinois | -3.12 | -12.28 to 6.04 | -0.02 | 1 |
| German Shepherd | -5.25 | -12.42 to 1.93 | -0.05 | 1 |
| Spanish Water Dog | -5.54 | -12.46 to 1.38 | -0.06 | 1 |
| Finnish Lapphund | -8.38 | -16 to -0.77 | -0.08 | 0.37 |
| Hovawart | -11.43 | -20.06 to -2.8 | -0.09 | 0.11 |
| Sex | 0.1 | -3.18 to 3.37 | 0.002 | 0.95 |
| Age | 0.1 | 0.03 to 0.18 | 0.09 | **0.009** |

**Supplementary Table 10.** Parameter estimates from the multiple regression for % of time (out of 120 s) spent on human-directed behaviour during the unsolvable task (n = 912), when completely independent dogs have been removed from the analysis. The Labrador Retriever was used as the reference breed. Breeds are ordered based on B-values, with breeds spending the most amount of time on human-directed behaviour at the top and breeds spending the least amount of time on human-directed behaviour at the bottom. P-values for breeds have been Bonferroni-corrected. Significant p-values are in bold.

| Variable | OR | 95 % CI | P-value |
| --- | --- | --- | --- |
| Breed |  |  |  |
| English Cocker Spaniel | 2.08 | 1.13 to 3.83 | 0.22 |
| Golden Retriever | 1.63 | 0.92 to 2.87 | 1 |
| Australian Kelpie | 1.5 | 0.72 to 3.16 | 1 |
| Mixed Breed | 1.49 | 0.93 to 2.37 | 1 |
| Finnish Lapphund | 1.4 | 0.75 to 2.59 | 1 |
| Hovawart | 1.39 | 0.69 to 2.77 | 1 |
| Shetland Sheepdog | 1.23 | 0.62 to 2.43 | 1 |
| Belgian Shepherd Malinois | 1 | 0.47 to 2.11 | 1 |
| German Shepherd | 0.94 | 0.53 to 1.69 | 1 |
| Spanish Water Dog | 0.84 | 0.46 to 1.52 | 1 |
| Border Collie | 0.67 | 0.39 to 1.16 | 1 |
| Australian Shepherd | 0.57 | 0.27 to 1.2 | 1 |
| Sex | 1.08 | 0.82 to 1.42 | 0.57 |
| Age | 0.99 | 0.98 to 0.99 | **<0.001** |

**Supplementary Table 11.** Parameter estimates from the logistic regression for the unsolvable task (n = 969), comparing dogs which abandoned the task (spent over 0 % of their time abandoning the task) to persistent dogs (those which spent 0 % of their time abandoning the task), with the Labrador Retriever as the reference breed. Breeds are ordered based on odds ratios, with breeds that are most likely to abandon the task at the top and breeds most likely to persist with the task at the bottom. P-values for breeds have been Bonferroni-corrected. Significant p-values are in bold.


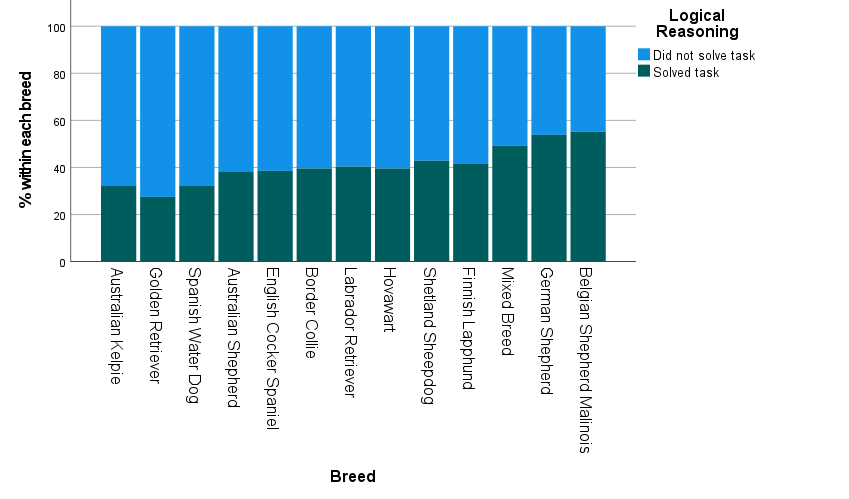

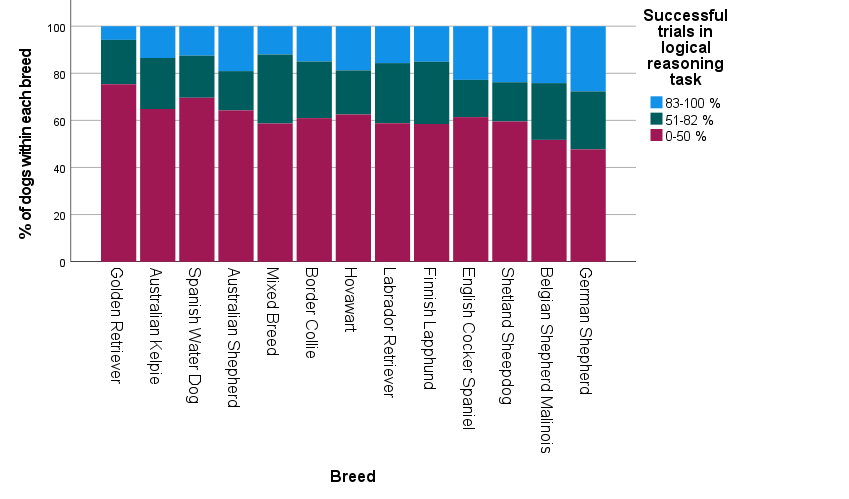


**a**

**b**

**Supplementary Figure 5**. Performance of breeds during the logical reasoning task (n = 826). a) Percentage of dogs within each breed which understood vs did not understand the logical reasoning task. Breeds have been ordered based on odds ratios, with breeds least likely to solve the task on the left and those most likely to solve the task on the right. b) Percentage of dogs within each breed having different success rates for the logical reasoning task. Breeds have been ordered based on odds ratios, with least successful breeds on the left and most successful breeds on the right.

| Variable | OR | 95 % CI | P-value |
| --- | --- | --- | --- |
| Breed |  |  |  |
| English Cocker Spaniel | 1.47 | 0.79 to 2.73 | 1 |
| Belgian Shepherd Malinois | 1.2 | 0.52 to 2.76 | 1 |
| Border Collie | 1.07 | 0.64 to 1.78 | 1 |
| Spanish Water Dog | 0.95 | 0.52 to 1.76 | 1 |
| Australian Shepherd | 0.89 | 0.45 to 1.78 | 1 |
| Golden Retriever | 0.85 | 0.48 to 1.53 | 1 |
| Finnish Lapphund | 0.65 | 0.34 to 1.23 | 1 |
| Shetland Sheepdog | 0.64 | 0.31 to 1.3 | 1 |
| Australian Kelpie | 0.62 | 0.28 to 1.37 | 1 |
| Mixed Breed | 0.52 | 0.29 to 0.93 | 0.32 |
| German Shepherd | 0.48 | 0.26 to 0.89 | 0.24 |
| Hovawart | 0.32 | 0.15 to 0.69 | **0.03** |
| Sex | 1.28 | 0.96 to 1.71 | 0.1 |
| Age | 1 | 1 to 1.01 | 0.7 |

**Supplementary Table 12.** Parameter estimates from the logistic regression for the gesture vs memory test (n = 823), with the Labrador Retriever as the reference breed. Breeds have been ordered based on odds ratios, with breeds most likely to trust the human gesture at the top and breeds most likely to trust their own memory at the bottom. P-values for breeds have been Bonferroni-corrected, and significant p-values are in bold.


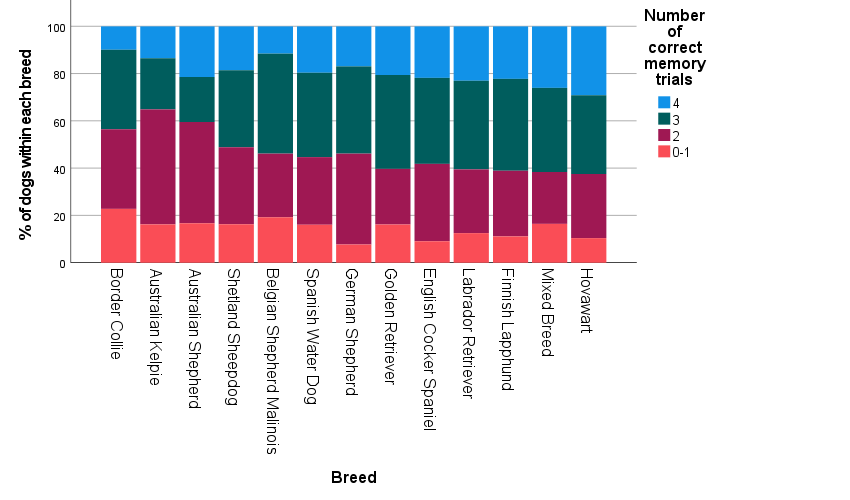


**Supplementary Figure 6.** Percentage of dogs within each breed with different success rates (number of correct trials out of 4) in the spatial memory task (n = 822), with the Labrador Retriever as the reference breed. Breeds have been ordered based on odds ratios, with least successful breeds on the left and most successful breeds on the right.

**References**

1 Tiira, K. & Lohi, H. Reliability and validity of a questionnaire survey in canine anxiety research. *Applied Animal Behaviour Science* **155**, 82-92 (2014).

2 *FitBark*, <<https://fitbark.com>>

3 Colpoys, J. & DeCock, D. Evaluation of the FitBark Activity Monitor for Measuring Physical Activity in Dogs. *Animals* **11**, 781 (2021).

4 Hall, C. & Ballachey, E. L. A study of the rat's behavior in a field. A contribution to method in comparative psychology. *University of California Publications in Psychology* (1932).

5 Walsh, R. N. & Cummins, R. A. The open-field test: a critical review. *Psychological bulletin* **83**, 482 (1976).

6 Carter, A. J., Feeney, W. E., Marshall, H. H., Cowlishaw, G. & Heinsohn, R. Animal personality: what are behavioural ecologists measuring? *Biological Reviews* **88**, 465-475 (2013).

7 Greggor, A. L., Thornton, A. & Clayton, N. S. Neophobia is not only avoidance: improving neophobia tests by combining cognition and ecology. *Current Opinion in Behavioral Sciences* **6**, 82-89 (2015).

8 Wormald, D., Lawrence, A. J., Carter, G. & Fisher, A. D. Validation of modified open field behaviour as a measure of trait anxiety in the dog. *Applied Animal Behaviour Science* **179**, 95-102 (2016).

9 Perals, D., Griffin, A. S., Bartomeus, I. & Sol, D. Revisiting the open-field test: what does it really tell us about animal personality? *Animal Behaviour* **123**, 69-79, doi:10.1016/j.anbehav.2016.10.006 (2017).

10 Dingemanse, N. J., Both, C., Drent, P. J., van Oers, K. & van Noordwijk, A. J. Repeatability and heritability of exploratory behaviour in great tits from the wild. *Animal Behaviour* **64**, 929-938, doi:10.1006/anbe.2002.2006 (2002).

11 Dingemanse, N. J. & Réale, D. Natural selection and animal personality. *Behaviour* **142**, 1159-1184 (2005).

12 Topál, J., Miklósi, Á., Csányi, V. & Dóka, A. Attachment behavior in dogs (Canis familiaris): a new application of Ainsworth's (1969) Strange Situation Test. *Journal of comparative psychology* **112**, 219 (1998).

13 Solomon, J., Beetz, A., Schöberl, I., Gee, N. & Kotrschal, K. Attachment security in companion dogs: Adaptation of Ainsworth’s strange situation and classification procedures to dogs and their human caregivers. *Attachment & human development* **21**, 389-417 (2019).

14 Kabadayi, C., Bobrowicz, K. & Osvath, M. The detour paradigm in animal cognition. *Animal cognition* **21**, 21-35 (2018).

15 Junttila, S., Huohvanainen, S. & Tiira, K. Effect of Sex and Reproductive Status on Inhibitory Control and Social Cognition in the Domestic Dog (Canis familiaris). *Animals* **11**, 2448 (2021).

16 Gacsi, M., Kara, E., Belenyi, B., Topal, J. & Miklosi, A. The effect of development and individual differences in pointing comprehension of dogs. *Anim Cogn* **12**, 471-479, doi:10.1007/s10071-008-0208-6 (2009).

17 Bray, E. E. *et al.* Early-emerging and highly heritable sensitivity to human communication in dogs. *Curr Biol* **31**, 3132-3136 e3135, doi:10.1016/j.cub.2021.04.055 (2021).

18 Reid, P. J. Adapting to the human world: dogs’ responsiveness to our social cues. *Behavioural processes* **80**, 325-333 (2009).

19 Kaminski, J. & Nitzschner, M. Do dogs get the point? A review of dog–human communication ability. *Learning and Motivation* **44**, 294-302 (2013).

20 Szetei, V., Miklósi, Á., Topál, J. & Csányi, V. When dogs seem to lose their nose: an investigation on the use of visual and olfactory cues in communicative context between dog and owner. *Applied Animal Behaviour Science* **83**, 141-152, doi:10.1016/s0168-1591(03)00114-x (2003).

21 Hauser, M. D., Comins, J. A., Pytka, L. M., Cahill, D. P. & Velez-Calderon, S. What experimental experience affects dogs’ comprehension of human communicative actions? *Behavioural processes* **86**, 7-20 (2011).

22 Riedel, J., Schumann, K., Kaminski, J., Call, J. & Tomasello, M. The early ontogeny of human–dog communication. *Animal Behaviour* **75**, 1003-1014, doi:10.1016/j.anbehav.2007.08.010 (2008).

23 Udell, M. A., Giglio, R. F. & Wynne, C. D. Domestic dogs (Canis familiaris) use human gestures but not nonhuman tokens to find hidden food. *Journal of comparative psychology* **122**, 84 (2008).

24 Wobber, V., Hare, B., Koler-Matznick, J., Wrangham, R. & Tomasello, M. Breed differences in domestic dogs’ (Canis familiaris) comprehension of human communicative signals. *Interaction Studies. Social Behaviour and Communication in Biological and Artificial Systems* **10**, 206-224, doi:10.1075/is.10.2.06wob (2009).

25 Bhattacharjee, D. *et al.* Free-ranging dogs are capable of utilizing complex human pointing cues. *Frontiers in psychology* **10**, 2818 (2020).

26 Tiira, K., Tikkanen, A. & Vainio, O. Inhibitory control–Important trait for explosive detection performance in police dogs? *Applied Animal Behaviour Science* **224**, 104942 (2020).

27 Mendes, J. W. W., Resende, B. & Savalli, C. A review of the unsolvable task in dog communication and cognition: comparing different methodologies. *Animal cognition* **24**, 907-922 (2021).

28 Brauer, J., Kaminski, J., Riedel, J., Call, J. & Tomasello, M. Making inferences about the location of hidden food: social dog, causal ape. *J Comp Psychol* **120**, 38-47, doi:10.1037/0735-7036.120.1.38 (2006).

29 Erdőhegyi, Á., Topál, J., Virányi, Z. & Miklósi, Á. Dog-logic: inferential reasoning in a two-way choice task and its restricted use. *Animal Behaviour* **74**, 725-737, doi:10.1016/j.anbehav.2007.03.004 (2007).

30 Aust, U., Range, F., Steurer, M. & Huber, L. Inferential reasoning by exclusion in pigeons, dogs, and humans. *Anim Cogn* **11**, 587-597, doi:10.1007/s10071-008-0149-0 (2008).

31 Stewart, L. *et al.* Citizen Science as a New Tool in Dog Cognition Research. *PLoS One* **10**, e0135176, doi:10.1371/journal.pone.0135176 (2015).

32 Chijiiwa, H. *et al.* Dogs and cats prioritize human action: choosing a now-empty instead of a still-baited container. *Anim Cogn* **24**, 65-73, doi:10.1007/s10071-020-01416-w (2021).

33 Petter, M., Musolino, E., Roberts, W. A. & Cole, M. Can dogs (Canis familiaris) detect human deception? *Behav Processes* **82**, 109-118, doi:10.1016/j.beproc.2009.07.002 (2009).

34 Dwyer, C. & Cole, M. R. Domesticated dogs (Canis familiaris) tend to follow repeated deceptive human cues even when food is visible. *Learn Behav* **46**, 442-448, doi:10.3758/s13420-018-0356-8 (2018).

35 Bhattacharjee, D. & Bhadra, A. Adjustment in the point-following behaviour of free-ranging dogs - roles of social petting and informative-deceptive nature of cues. *Anim Cogn*, doi:10.1007/s10071-021-01573-6 (2021).

36 Fiset, S., Beaulieu, C. & Landry, F. Duration of dogs' (Canis familiaris) working memory in search for disappearing objects. *Anim Cogn* **6**, 1-10, doi:10.1007/s10071-002-0157-4 (2003).
